# Ultrafast Spectroscopy of Commercial Sunscreen Formulations in Thin Films and on a Skin Mimic

**DOI:** 10.1021/acsomega.5c12445

**Published:** 2026-03-11

**Authors:** Abigail L. Whittock, Jack M. Woolley, Juan Cébrian, Vasilios G. Stavros, Natércia d. N. Rodrigues

**Affiliations:** † Department of Chemistry, 2707 University of Warwick, Gibbet Hill Road, Coventry CV4 7AL, U.K.; ‡ Department of Physics, University of Warwick, Gibbet Hill Road, Coventry CV4 7AL, U.K.; § Warwick Centre for Ultrafast Spectroscopy, University of Warwick, Coventry CV4 7AL, U.K.; ∥ The Lubrizol Corporation, 504741 Lipotec SAU, Calle Isaac Peral, 17 Pol. Ind. Camí Ral, Barcelona 08850, Spain; ⊥ School of Chemistry, 1724 University of Birmingham, Birmingham B15 2TT, U.K.; # iBB-Institute for Bioengineering and Biosciences, Instituto Superior Técnico, 72971 Universidade de Lisboa, Lisboa 1049-001, Portugal

## Abstract

The photodynamics of a given chromophore are usually affected by the surrounding environment (e.g., solvent or matrix). As a result, it is often assumed that the ‘fundamental’ spectroscopic behavior of a given chromophore, usually studied in dilute solution, is not relevant to the understanding of more complex mixtures. In this work, we demonstrate that the ‘fundamental’ photodynamics of common sunscreen UV filters are comparable to those observed in formulation, including the complex interactions between them, and even for formulation applied to a skin mimic. In particular, we have carried out ultrafast laser spectroscopy studies directly on sunscreen formulations containing methyl anthranilate (MA), ethylhexyl methoxycinnamate (EHMC), and octocrylene (OCR) as commonly used UV filters. Our results not only have significant implications for the sunscreen industry, providing direct evidence of UV filter interactions in formulation, but importantly they are demonstrative of the relevance of ‘fundamental’ spectroscopic techniques to understanding real-life systems.

## Graphical abstract

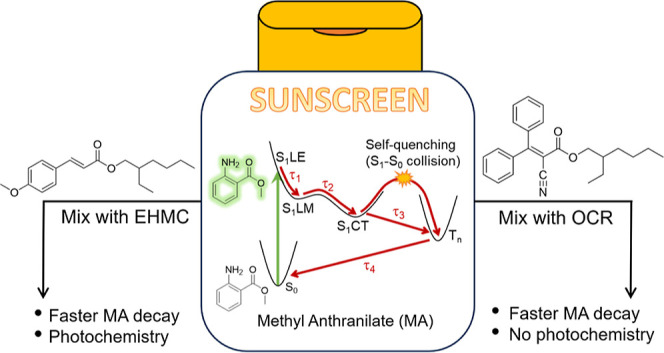

## Introduction

The ultraviolet (UV) radiation that reaches the Earth from the Sun, commonly subdivided into UVB (280–320 nm) and UVA (320–400 nm), can cause damage to living organisms.^1^ Plants and animals both have their own natural mechanisms to avoid this photodamage.^2–5^ In humans, photoprotection is afforded by melanin, a pigment produced in human skin which absorbs UV radiation precluding it from reaching more vulnerable skin cells.^4^ However, this natural photoprotection is not always sufficient, and sunburn can nevertheless occur as a result of photodamage to the skin. It is now commonplace, and indeed highly recommended, that photoprotective lotions, i.e. sunscreens, are applied to the skin on a regular basis, particularly when prolonged Sun exposure is unavoidable.^6^

The active ingredients present in sunscreen products are UV filters which absorb the harmful UV radiation to avoid photodamage to the skin. When absorbing UV radiation, these filters are photoexcited to a more energetic (called excited) electronic state, and this excess energy must then be dissipated over time. Ideally, a sunscreen UV filter would dissipate excess energy within a femto- to picosecond time scale (10^–15^ to 10^–12^ seconds, fs to ps, respectively) to avoid molecular disintegration or any other potentially harmful side chemistry caused by energy transfer to the sunscreen formulation.^5,7^

Over the past few years, the mechanisms by which sunscreen UV filters dissipate absorbed energy—commonly referred to as a molecule’s photodynamics—have been the focus of comprehensive studies, the results of which have been thoroughly reviewed.^5,7–10^ Despite recent efforts to study sunscreen UV filters in more realistic media, most spectroscopic studies on sunscreen UV filters have been undertaken in single-solvent mixtures. These studies have revealed not only that the photodynamics of sunscreen UV filters are often significantly impacted by environmental conditions such as solvent polarity, viscosity, or pH, but also that the way in which a given factor affects the observed photodynamics is system specific.^7,11–13^

In commercially available sunscreens, UV filters are incorporated in complex formulations, which raises questions regarding the relevance of so-called ‘fundamental’ (i.e., single-solvent) spectroscopic studies to the development of commercial sunscreen products. In the work presented here, we aimed to establish if and how the photodynamics observed in formulation differ from those previously observed in single solvent mixtures, thus exploring the suitability of fundamental spectroscopic methods to inform commercial sunscreen development. Importantly, we explore not only the photodynamics of each UV filter individually, but also the interactions between them, to ascertain if and how these interactions are impacted by the complex formulation mixture.

We have carried out transient electronic absorption spectroscopy (TEAS) studies directly on commercial sunscreen formulations, prepared according to pre-established commercial formulas, both in the form of thin solid films on an inert surface (calcium fluoride windows, CaF_2_) and applied to a skin mimic. The complex formulations studied in this work included methyl anthranilate (MA, see Figure 1 for molecular structure) on its own or in combination with other commercially available UV filters, namely ethylhexyl methoxycinnamate (EHMC) and octocrylene (OCR), the molecular structures of which are shown in Figure 1. Our results find important similarities between the photodynamics of each of these UV filters in solution and in formulation; we also find evidence of a quenching mechanism observed between MA + EHMC and MA + OCR in dilute solutions, which persists (and is potentially enhanced) in formulation. These observations are important to the sunscreen industry and, more broadly, are demonstrative of the relevance of ‘fundamental’ spectroscopic knowledge to the understanding of real-life systems and the development of novel solutions to current challenges.

**Figure 1:**
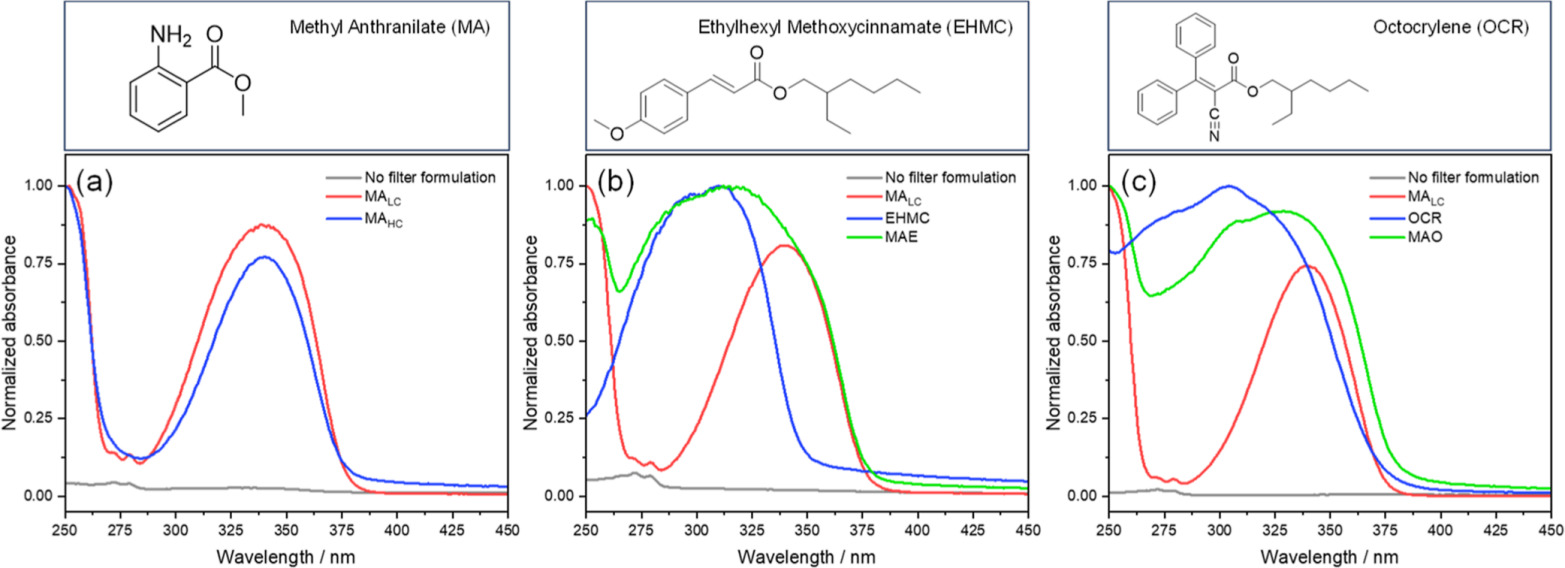
(Top) molecular structures of methyl anthranilate (MA), ethylhexyl methoxycinnamate (EHMC), and octocrylene (OCR). (Bottom) UV/vis spectra for thin solid films (of variable thickness, see methods for further details) of the formulations under study in this work. In all cases, the gray line represents the absorption spectra of the ‘reference’ formulation, prepared with no UV filters but all other ingredients (see Section S1 of the Supporting Information for complete formulas). The graphs show comparisons between (a) formulations MA_LC_ (red line) and MA_HC_ (blue line); (b) formulation MA_LC_, E (denoted EHMC), and MAE; and (c) formulation MA_LC_, O (denoted OCR), and MAO.

## Results and Discussion

### Formulations in Thin Solid Films

The formulations under study in the present work were prepared according to procedures described in a previous publication;^14^ the complete list of ingredients in each formulation, as well as an overview of the preparation procedure, can be found in Section S1 of the Supporting Information, in Tables S1 and S2. The samples under study in this work consist of typical commercial sunscreen formulations, adapted to include the UV filters of interest. Two of these formulations include only MA, at different concentrations: MA_LC_ is the formulation with the lowest concentration of MA, i.e. 4% (w/w), while MA_HC_ contains a higher concentration of MA, at 19.5% (w/w); this last concentration is much higher than the maximum concentration of MA permitted in commercial sunscreen regulations (5%),^15^ but we use it here to highlight the extreme case. The study of the highest concentration formulation also serves to ascertain whether any differences in photodynamics observed in formulation (vs dilute solution) can be attributed to the complex environment or to concentration effects. Combination formulations were also prepared, for which 9.80% (w/w) of MA is combined with 9.70% (w/w) of EHMC (referred to as MAE) or OCR (MAO). The concentration of MA in these combination formulations is also higher than that allowed in commercial sunscreens, but we prioritized using similar concentrations of each UV filter in the mixture to avoid the photodynamics of either one of them overwhelming the other. In addition, to verify whether, individually, EHMC and OCR behave in formulation as observed in neat solvent solutions,^16,17^ we prepared formulations containing only EHMC (formulation E) or OCR (formulation O), in each case at 9.70%(w/w) (see Table S2). The results and discussion of TEAS measurements on formulations E and O can be found in Section S2 of the Supporting Information. Finally, to establish the instrument response of our experiments, we prepared a formulation containing no UV filters (see Section S3 of Supporting Information for further details and measurements which place the instrument response at ∼60 fs).

Thin solid films of each sunscreen formulation were prepared by pressing a small amount of the formulation between two calcium fluoride (CaF_2_) windows, which were translated perpendicularly to the incoming laser beams during experiments to avoid sample photobleaching (see Methods for further details). The samples thus prepared were irradiated with laser pulses at 335 nm, the peak absorbance of MA according to previous studies^16–18^ and as evident from the spectra in Figure 1. Nevertheless, 335 nm also significantly photoexcites EHMC and OCR in mixed formulations (see Figure 1). The transient absorption spectra (TAS) obtained for each of the formulations studied are presented in Figures 2 and 3. The time constants extracted from these data sets are given in Table 1.

**Figure 2:**
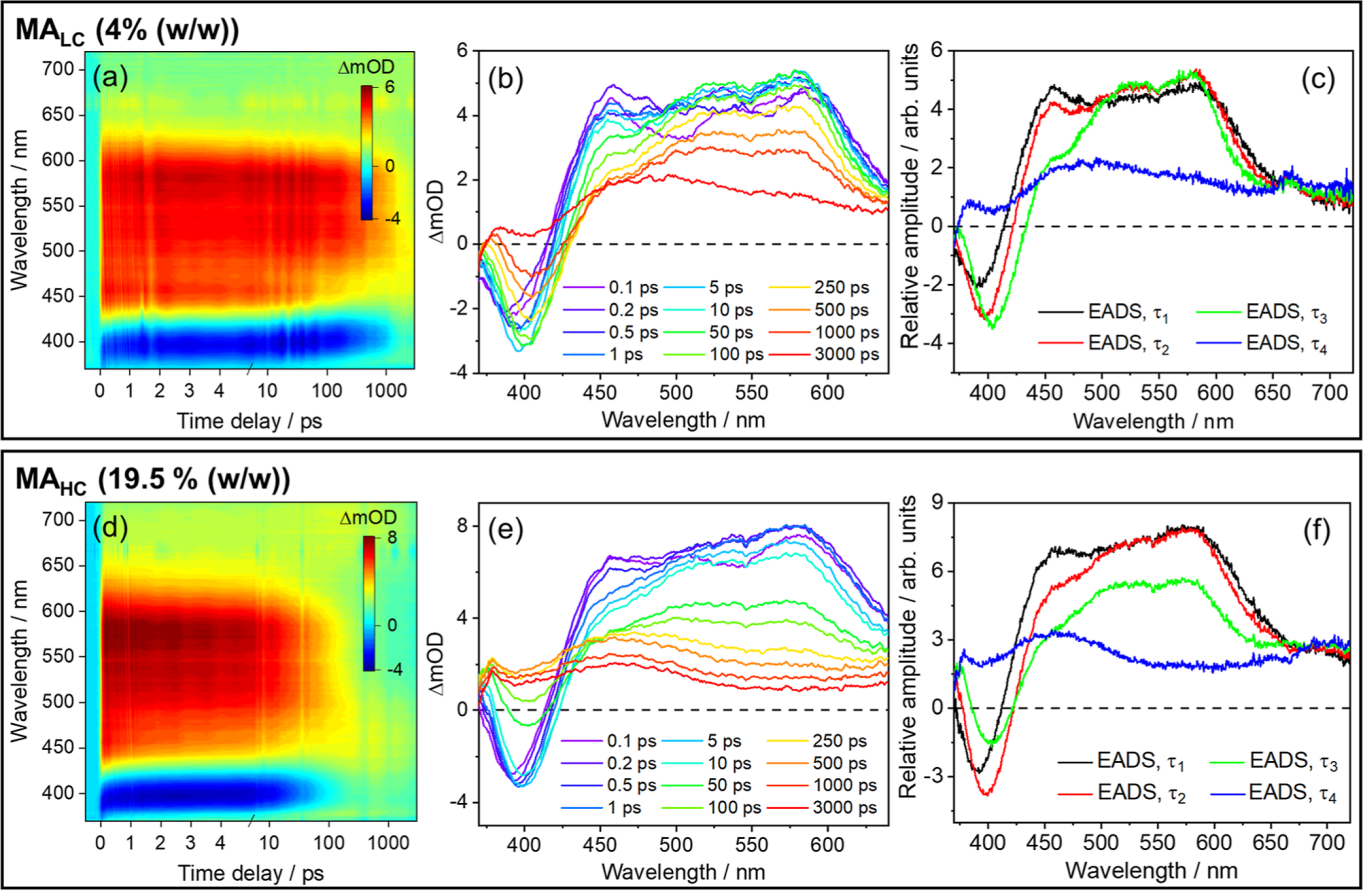
TEAS results for formulations MA_LC_ (MA at 4% (w/w), top) and MA_HC_ (MA at 19.5% (w/w), bottom). In both cases, samples were photoexcited at λ_pu_ = 335 nm. TEAS data are presented as a false color heatmaps in (a,d), with time being plotted linearly until 5 ps and on a logarithmic scale from then on. The same data are presented as transient absorption spectra at selected pump–probe time delays (Δ*t*) in (b,e). Finally, (c,f) show the EADS associated with each of the time constants (τ), as extracted from globally fitting the corresponding TEAS data.

**Figure 3:**
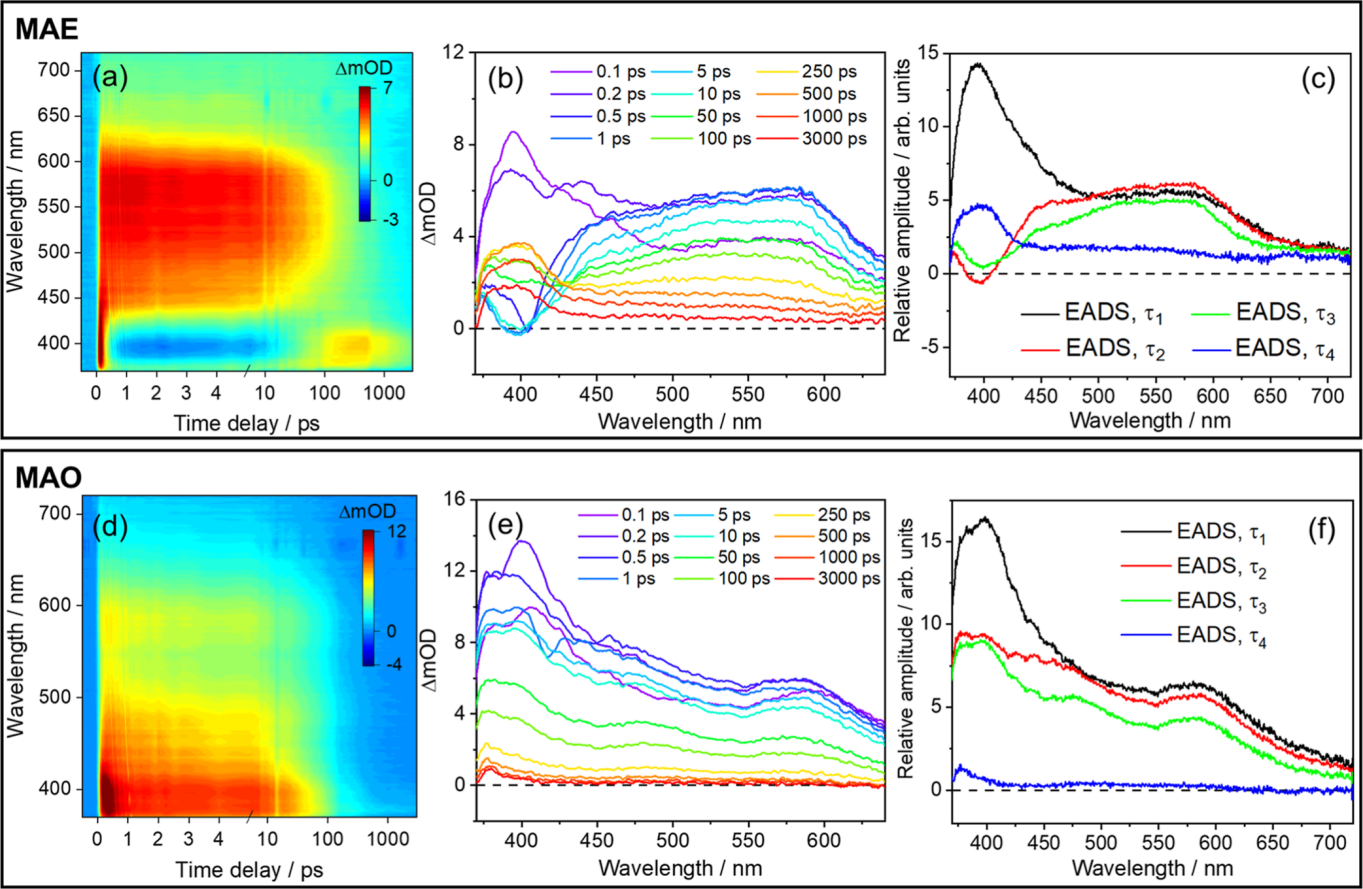
TEAS results for formulations MAE (MA and EHMC mixture, top) and MAO (MA and OCR mixture, bottom). In both cases, samples were photoexcited at λ_pu_ = 335 nm. TEAS data are presented as a false color heatmaps in (a,d), with time being plotted linearly until 5 ps and on a logarithmic scale from then on. The same data are presented as transient absorption spectra at selected pump–probe time delays (Δ*t*) in (b,e). Finally, (c,f) show the EADS associated with each of the time constants, as extracted from globally fitting the corresponding TEAS data.

**Table 1:** Time Constants Extracted in the Present Work from the TEAS Data of Each of the Formulations under Study^a^

time constant	MA_LC_	MA_HC_	MAE	MAO	MA in cyclohexane^18^	MA in methanol^18^
τ_1_/fs	900 ± 60	760 ± 60	180 ± 60	380 ± 60	-	-
τ_2_/ps	61 ± 2	15.2 ± 0.5	7.8 ± 0.2	4.3 ± 0.1	4.6 ± 0.2	5.9 ± 0.2
τ_3_/ps	830 ± 23	128 ± 3	112 ± 2	92 ± 0.7	>2000	>2000
τ_4_/ns	>3	>3	>3	2 ± 0.03	>2	-

a The errors quoted are either those associated with the fit, as produced by the fitting software package (reported to two standard deviations) or the instrument response, whichever is largest; more details can be found in the methods section. For comparison, the time constants reported in Rodrigues *et al.* ^18^ for MA in cyclohexane and methanol (10^–3^ M, λ_pu_ = 330 nm) are also shown.

We start by discussing the results obtained for MA_LC_, the formulation containing MA at the lowest concentration, i.e., 4% (w/w). It is worth noting that this is still a much higher concentration than what is typical for previously studied solutions of MA (10^–3^ M, i.e. ≪1% (w/w)).^18^ The TAS obtained for MA_LC_, shown in Figure 2a,b, are similar to those obtained in solution:^18^ immediately after photoexcitation, the TAS consist of a stimulated emission (SE) feature at 385 nm and a broad excited state absorption (ESA) feature which extends for the remainder of the probe window, i.e. between 450 and 650 nm. These features are most clearly perceptible in the evolution associated difference spectra (EADS, see Methods) shown in Figure 2c. Within τ_1_ = 900 ± 60 fs (see Table 1), as the EADS evolve from black to red in Figure 2c, the intensity of the SE feature increases slightly as its peak red-shifts toward 400 nm. A slight decay of the broad ESA is also discernible on this time scale. This behavior is typical of an initial geometry rearrangement as a result of vibrational cooling of the photoexcited molecule. Considering previously published computational studies, which provide insight into the potential energy landscape of MA,^18^ this vibrational cooling would occur as the excited state population moves away from the Franck–Condon (locally excited, S_1_LE) region toward an energy minimum on the S_1_ surface, likely alongside some solvent rearrangement.

In previous studies into MA’s photodynamics in solution,^18^ a single time constant of 2–11 ps (depending on solvent) is assigned to vibrational energy transfer as the excited population moves from the Franck–Condon region (S_1_LE) to the S_1_ charge transfer minimum (S_1_CT).^18^ In formulation, there appears to be further movement away from S_1_LE, captured by the second time constant extracted, τ_2_ = 61 ± 2 ps (see Table 1) and associated with a further red shift of the SE feature, alongside a more noticeable decay of the ESA feature (as the red EADS evolves to green, see Figure 2c). As such, we propose that the effect of the formulation matrix might create an additional local energy minimum along the S_1_LE → S_1_CT path (henceforth referred to as S_1_LM), that in solution is either not present or too shallow to trap excited state population. In the absence of this proposed local minimum, the solution environment would allow for a faster evolution of excited state population from S_1_LE to S_1_CT within 2–11 ps, while in the formulation environment the same movement takes place in two steps, i.e. S_1_LE → S_1_LM within τ_1_ ∼900 fs, and then S_1_LM → S_1_CT within τ_2_ ∼62 ps.

After the initial S_1_LE $\overset{\tau_{1}}{\to }$ S_1_LM $\overset{\tau_{2}}{\to }$ S_1_CT migration, as just proposed, we see the system evolve within τ_3_ = 830 ± 23 ps (from the green to blue EADS, see Figure 2c) toward a drastically different spectrum where a broad ESA centered at ∼470 nm is visible, spectrally similar to what has previously been assigned to MA’s triplet state transient absorption;^18,19^ for comparison with the data in Figure 2, complementary TAS and EADS for MA in dilute solution are presented in Section S4 of the Supporting Information. Given these similarities, we propose that the broad ESA in the long-time TEAS and EADS (see Figure 2) correspond to the transient absorption of MA’s triplet state, and τ_3_ is therefore assigned to intersystem crossing (ISC), as excited state population evolves from the S_1_ state into the accessible triplet state.

The equivalent time constant in dilute solution is significantly longer (>2 ns).^18^ As discussed in further detail in Section S4 of the Supporting Information, we argue that ISC is faster in formulation than in solution due to self-quenching of MA,^14^ an intermolecular process by which molecular collisions facilitate energy dissipation. Self-quenching has previously been observed in Meradimate,^20^ a close analogue of MA. Further to this, according to Erickson’s methodology,^21^ the approximate distance between two MA molecules in a 10^–3^ M solution is 118 Å, compared to 18 Å in formulation MA_LC_ and 11 Å in MA_HC_; the order of magnitude shorter intermolecular distance in formulation allows efficient intermolecular energy transfer,^22^ which further supports the hypothesis of self-quenching being favored in formulation, and more generally in samples with higher concentration of MA.

Specifically, we propose a Dexter (collisional) quenching mechanism: collisions between two MA molecules, one in the S_1_ and one in the ground state, result in energy transfer that quenches the molecule in the S_1_ state (making it return to the ground state) and simultaneously promote the molecule in the ground state into the S_1_ state, from which ISC onto the triplet state can then also take place. Higher concentrations encourage collisions; hence, we see both faster S_1_ quenching and faster generation of triplet states in samples with higher concentration of MA (dilute solution < MA_LC_ < MA_HC_). The faster generation of triplet states comes as a result of the two pathways at play in formulation: directly from photoexcited MA, and from the self-quenching mechanism, as just described. We note here that singlet–singlet annihilation could also possibly facilitate the faster decay of the S_1_ state of MA. However, singlet–singlet annihilation would not result in faster generation of triplet states, which is evidenced in our data by the earlier appearance of the ESA attributable to the triplet state of MA in MA_HC_. As such, while we cannot conclusively exclude singlet–singlet annihilation, we do not consider this to be the dominant decay pathway in formulations with high concentration of MA.

To add, finally, no ingredient in the formulations under study contains any heavy atoms (such as halogens or transition metals) that may influence the rate of ISC. We carried out equivalent TEAS measurements for the reference formulation, containing no UV filters but all other ingredients (see Section S1 of the Supporting Information, namely Tables S1 and S2 for complete formulas), having found that it yields no discernible dynamics apart from time-zero artifacts (see Section S3 of the Supporting Information).

Finally, the last time constant extracted from TEAS measurements of MA_LC_, τ_4_ > 3 ns, extends beyond the temporal window of these experiments and is assigned to the triplet state lifetime for MA in formulation. Unlike what was observed in solution, there seems to be a broad absorption underlying the triplet state absorption feature (since the baseline is nonzero), covering the whole probe window and indeed with a peak at ∼375 nm. This effect could result from either a photoproduct and/or MA’s interaction with other components in the formulation; from previous studies we know MA undergoes considerable photodegradation upon irradiation with simulated solar light^23^ hence the occurrence of a photoproduct is entirely plausible. An analogous feature is not visible in equivalent data for the ‘reference’ formulation, containing no UV filters (see Figure S3 in Section S3 of the Supporting Information).

Inspection of the corresponding data for the MA_HC_ formulation, containing a higher concentration of MA (i.e., 19.5% (w/w) c.f. 4% (w/w) in MA_LC_), reveals very similar TAS, with mostly the same spectral features and time-dependent behaviors as what was just described for MA_LC_ (see Figure 2). As before, the SE feature increases in intensity and spectrally red-shifts, and the ESA feature decays as the EADS evolve from black to red (see Figure 2f) within τ_1_ = 760 ± 60 fs. This time constant, again assigned to the migration of excited state population from S_1_LE to S_1_LM, is shorter than its equivalent in MA_LC_ (∼700 fs vs ∼900 fs, respectively), which suggests that excited state relaxation is slightly faster for the formulation with a higher concentration of MA. As discussed earlier, the faster excited state decay in higher concentration formulations can be rationalized by considering a self-quenching mechanism in MA.^14^

Further evidence for this accelerated excited state decay in MA_HC_ comes from the evolution of the EADS associated with τ_2_ (from red to green, see Figure 2f): not only the decay of the ESA feature (450–650 nm) is much more pronounced here than the corresponding process in MA_LC_, but also a decrease in intensity of the SE feature is now observed, rather than the continued increase seen in MA_LC_. In isolation, either one of these observations could indicate environment-induced alterations to the excited state landscape of MA, resulting in poorer overlap or weaker oscillator strength between states. Taken together, however, we argue that these observations are more likely indicative of faster excited state relaxation brought about by self-quenching. It is worth noting, however, that at this point we still consider the excited state population to be in the S_1_ state, given the persistence of the initially observed spectral features. Hence, we assign τ_2_ = 15.2 ± 0.5 ps to migration of excited state population from S_1_LM to S_1_CT, as before; once again this time constant is faster than its equivalent in the lower concentration formulation.

The time constant equivalent to the one previously assigned to ISC is also much faster in MA_HC_, at τ_3_ = 128 ± 3 ps (c.f. τ_3_ = 830 ± 23 ps in MA_LC_), again in line with our previous argument for faster ISC in higher concentration samples. However, we cannot discard that other factors contribute to this marked difference; for example, the combination of S_1_ relaxation pathways—fluorescence, self-quenching and any proportion of internal conversion that may take place—may become rate-limiting, contributing to a shorter ISC time constant. It is also possible that the high concentration environment generates sufficient intermolecular interactions to alter the relative energies of the excited states, ultimately resulting in a more energetically accessible singlet–triplet crossing, and thus faster ISC in MA_HC_.

Finally, as before, the final EADS for MA_HC_ (see Figure 2f) closely resembles the previously reported transient absorption of the triplet state of MA, and therefore τ_4_ > 3 ns is assigned to this triplet state lifetime. The baseline underlying the observed triplet state absorption feature is nonzero in this case also, again suggesting a photoproduct and/or MA’s interaction with other components in the formulation. We recall that no analogous feature is visible in equivalent data for the ‘reference’ formulation, containing no UV filters (see Section S3 of the Supporting Information).

Before moving on to discuss the results obtained for formulations containing mixtures of MA with either EHMC or OCR (MAE and MAO, respectively), we note here that we have carried out TEAS measurements for formulations whose only UV filters were either EHMC or OCR, to ascertain whether the behavior they each exhibit in solution is maintained in formulation. As discussed in further detail in Section S2 of the Supporting Information, we find that the individual behavior of both EHMC and OCR in formulation closely resembles observations in solution, in terms of both spectral features and their evolution within the typical time window of these experiments. Considering these results, we conclude that the photodynamic behavior observed in the formulations containing mixtures of MA and either EHMC or OCR is due to the interaction between UV filters, and does not result from any effects of the complex formulation environment on the UV filters individually (see Section S2 of the Supporting Information for further discussion).

Immediately after photoexcitation of MAE, a broad ESA between ∼450 and 650 nm is observed which can clearly be assigned to MA (see Figure 3a,b). An additional ESA feature, stronger and centered at 400 nm, is also visible, clearly matching observations for EHMC in solution^16^ and in formulation (see Figure S1 in Section S2 of the Supporting Information). In early times (Δ*t* < 1 ps), the intensity of the ESA feature from EHMC is sufficient to drown the SE feature previously seen in formulations containing only MA (MA_LC_ and MA_HC_). Within τ_1_ = 180 ± 60 fs (as the black EADS evolves to red in Figure 3c), the ESA feature attributable to EHMC disappears, leaving behind only MA-like features. This is spectrally and temporally consistent with what is observed in EHMC-only formulations, for which the strong ESA at ∼400 nm completely disappears in approximately 1 ps, leaving almost no other visible features (apart from a weak, broad ESA between ∼500 and 600 nm, see Figure S1 in Section S2 of the Supporting Information). As such, in the case of MAE, τ_1_ is primarily assigned to the photoisomerization process in EHMC, as both the spectral changes and time scales are reminiscent of what has previously been observed for EHMC.^16^ Nevertheless, τ_1_ will inevitably encompass (apparently minor) contributions from MA and solvent photodynamics.

After the decay of the EHMC feature, the spectra (and EADS, see Figure 3b,c, respectively) consist of features clearly attributable to MA, including the SE feature at 400 nm, and the broad ESA in the 450–650 nm region. There is then a decay of both features (as the red EADS evolves into green, see Figure 3c), within τ_2_ = 7.8 ± 0.2 ps, which as before we assign to excited state relaxation of MA, presumably as excited state population migrates toward S_1_CT (see earlier discussion). We note that this process is faster in this formulation containing a mixture of MA and EHMC when compared to either MA_LC_ or MA_HC_ (i.e., MA-only formulations, regardless of concentration), which could be indicative of EHMC’s quenching effect on MA.

In previous work, Matsumoto *et al.* had already reported on the efficient triplet–triplet energy transfer between MA and EHMC, the rate constant of which the authors reported to be approximately 10^10^ mol^–1^ dm^3^ s^–1^ (with moderate dependence on solvent).^19^ At the concentrations we are working with in our formulations, we would therefore expect this triplet–triplet energy transfer to occur with a time constant of hundreds of picoseconds (∼600 ps), which we do not observe. The authors further mention that singlet–singlet energy transfer would be energetically unfavorable between MA and EHMC (with EHMC acting as the quencher), since the energy of the S_1_ state of EHMC is higher than that of MA.^19^

Nevertheless, the EADS associated with τ_2_ for formulation MAE (see Figure 3c) do show a faster decay of spectral features attributable to MA than that observed in the absence of EHMC (see Table 1). Further evidence of the faster decay of spectral features attributable to MA in the presence of EHMC can be gathered by comparing the TAS for formulation MAE with the sum of TAS for formulations containing MA and EHMC separately (no other data processing other than the sum; see Section S5 of the Supporting Information for further details). This comparison clearly shows that, up to 10 ps, the TAS for formulation MAE is fairly similar to the sum of the TAS for MA and EHMC formulations. After that (at 50, 500, and 2500 ps, see Figure S6 in Section S5 of the Supporting Information), the TAS for formulation MAE are no longer sufficiently described by the additive behavior of each UV filter separately, and the features attributable to MA disappear much faster, further compounding the evidence for an interaction taking place between MA and EHMC.

Evidence of the nature of this interaction can be gathered from the last EADS for formulation MAE (blue line in Figure 3c), where a strong, broad ESA centered at ∼400 nm is visible, with a significant baseline signal. A corresponding feature can clearly be seen in the TAS for formulation MAE from Δ*t* = 250 ps, with evidence for its growth from as early as Δ*t* = 10–50 ps, when the SE feature attributable to MA disappears and steadily gives rise to this new ESA feature at ∼400 nm. This ESA feature cannot be assigned to the *Z* isomer previously found to result from the photoisomerization of EHMC,^16^ since that isomer yields an ESA centered at ∼350 nm. Furthermore, the TAS for formulations containing MA or EHMC individually do not show a comparable ESA at ∼400 nm (see Figures 2 and S1, respectively), hence we conclude that this ESA feature, observed only in TAS for the mixture formulation MAE, is likely due to photochemistry taking place between MA and EHMC. We have no conclusive evidence to determine whether this photochemistry would involve either excited singlet or triplet states of either molecule, but the UV/vis spectra in Figure 1 suggest no ground state complex is formed between MA and EHMC. The absorption at 400 nm should therefore correspond to an unidentified photoproduct generated by interaction of excited states of MA and EHMC. We note here, however, that the ESA feature centered at 400 nm seems to decay in the time from when it first appears, at approximately 250 ps. While this could be an ‘artificial’ decay, made apparent only due to the overall decay of other overlapping spectral features, we cannot discard that the ‘photoproduct’ formed would in fact an exciplex, i.e. an excited state complex between two different molecules (in this case, MA and EHMC) that appears upon photoexcitation but disappears as excess energy is dissipated. This is a relatively common process;^24^ an exciplex might explain the lack of evidence in previous studies for a ‘steady-state’ photoproduct resulting from the interaction between MA and EHMC.^23^ Finally, we further note that while there are techniques that could in principle detect and identify any photoproducts resulting from irradiation of these formulations, such as the ones employed in Rioux *et al.*’s work,^25^ for example, this would necessitate further, more comprehensive studies which are beyond the scope of the present work.

We hypothesize that quenching of MA by EHMC is probably not the primary reason for the slightly faster decay of MA in the presence of EHMC. Given the evidence just discussed, it is possible that the faster decay of MA in formulation MAE results from the consumption of MA in the reaction with EHMC, which yields the photoproduct (or exciplex) with an ESA feature at ∼400 nm. Nevertheless, we cannot discard the possibility that the high concentrations of the formulations under study may facilitate energy transfer mechanisms that lead to quenching of MA’s excited state; in these formulations, the distance between molecules is estimated at 10–20 Å (much less than for the dilute solutions studied in Matsumoto *et al.*’s work),^19^ which may allow Dexter quenching and thus further explain the faster decay of MA spectral features in the presence of EHMC. As such, we propose that τ_3_ = 112 ± 2 ps in formulation MAE encompasses a combination of processes, including contributions from quenching of MA by EHMC, the generation of a photoproduct resulting from photochemistry between MA and EHMC, and ISC in MA. Finally, τ_4_ > 3 ns in formulation MAE is proposed to correspond to the lifetimes of the MA + EHMC photoproduct (ESA at ∼400 nm) and the triplet state of MA (the broader ‘baseline’ signal in the 450–650 nm region).

Finally, we discuss the results obtained for MAO, a sunscreen formulation containing both MA and OCR, the latter of which is a known quencher of other UV filters.^26^ Once again, the TAS obtained for this formulation seems to include elements that can be attributed to both MA and OCR. Namely, the strong ESA between 350 and 450 nm matches previous observations for dilute solutions of OCR,^17^ as well as the TAS obtained for formulation O, containing only OCR as the UV filter (see Figure S2 in the Supporting Information). In addition, there is a broader ESA across the red edge of the probe window (450–650 nm) which can be attributed to MA, with significant overlap with the ESA from OCR. This overlap also results in the disappearance of the SE feature present in the TAS for MA-only formulations.

The photodynamics previously reported for OCR in dilute solution indicate an ultrafast decay of excited state population. Specifically, Baker *et al.* ^17^ proposed that initial photoexcitation of OCR (at λ_pu_ = 300 nm) would populate an ensemble of close in energy *n* ^1^ππ* states (*n* ≥ 1) which would subsequently decay to lower lying excited states, with internal conversion (IC) back onto the vibrationally hot S_0_ state eventually taking place within 180–200 fs. The vibrational cooling of the S_0_ state would then be completed by approximately 1.5 ps in methanol (0.8 ps in cyclohexane).^17^ As further discussed in Section S2 of the Supporting Information, these ultrafast photodynamics are generally retained for OCR in formulation, despite less evident vibrational relaxation, with IC taking place in approximately 400 fs. The TAS for OCR in formulation also suggest some formation of a photoproduct, which is not observed in solution (see Section S2 of the Supporting Information for further details, particularly Figure S2).

For formulation MAO, containing a mixture of MA and OCR, the first time constant extracted from the TAS (τ_1_ = 380 ± 60 fs) can be assigned to the initial decay of excited state population in both OCR and MA, as previously discussed. The EADS corresponding to this process (from black to red in Figure 3f) show simply a decay of all ESA features (more pronounced for the ESA attributable to OCR, i.e. 350–450 nm), which is consistent with excited state relaxation. Afterward, as the EADS evolve from red to green in Figure 3f within τ_2_ = 4.3 ± 0.1 ps, there is a further decay of ESA features, but this time more pronounced in the wavelength range attributable mostly to MA, which suggests τ_2_ to be related mostly to the S_1_LM → S_1_CT relaxation described earlier for MA. The next EADS, corresponding to τ_3_ = 92 ± 0.7 ps, show an almost complete decay of all observed spectral features (as the green EADS evolves to blue in Figure 3f), apart from a small ESA at ∼375 nm which persists for the remainder of the temporal window of these experiments, i.e. τ_4_ > 3 ns. This longer-time behavior is once again significantly different from what is observed in formulations containing only MA, again suggesting that the interaction with OCR results in faster decay of MA’s excited states.

Following the same approach as before, we compared the TAS data for formulation MAO with the added spectra for formulations MA_LC_ and O (containing only MA and OCR as the UV filters, respectively) at given pump–probe time delays, to ascertain the origins of this apparent quenching. Unlike the case for EHMC, the evidence of interaction between MA and OCR is immediately present (see Figure S7 in the Supporting Information): even at Δ*t* = 0.5 ps, adding the TAS for MA and OCR does not satisfactorily reproduce the equivalent spectrum for the MAO formulation, with the large ESA at 350–450 nm standing out as the main difference. While this ESA feature somewhat resembles the one observed for OCR, we note that this feature has completely disappeared by Δ*t* ∼10 ps in formulation O (see Figure S2 in the Supporting Information), while in MAO the analogous feature can be seen as late as Δ*t* ∼100 ps (see Figure 3e), clearly indicating an interaction between MA and OCR. In addition, the TAS for MAO are drastically different from the MA + OCR added spectra at all values of Δ*t* analyzed (see Figure S7 in the Supporting Information), not only due to the persistence of the aforementioned ESA at ∼350–450 nm, but also due to the rapid decay of the any spectral features attributable to MA. Finally, similar to the case of the MAE mixture, a small ESA appears at later Δ*t*, this time at approximately 375 nm. However, unlike the MAE case, this feature matches observations both in the added spectra and in the TAS for MA-only formulations, and can therefore be attributed to MA-only photodynamics. As such, in the case of MAO we have strong evidence for interaction between MA and OCR, faster decay of MA, and no generation of photoproducts.

In light of the above discussion (and also the discussion in Section S5 of the Supporting Information), we assign τ_3_ = 92 ± 0.7 ps to quenching of MA by OCR, likely facilitated by the close proximity of molecules in these high concentration formulations (c.f. dilute solutions, see earlier discussion). While our data undoubtedly reveals this interaction between MA and OCR, singlet-to-singlet energy transfer between these molecules is unfavorable, since the S_1_ state of OCR is higher in energy than that of MA, as previously pointed out by Matsumoto *et al.* ^19^ As such, we propose that quenching of MA by OCR occurs via one of two potential mechanisms. One option would be triplet-to-triplet energy transfer, however, this seems unlikely, since we have evidence for the triplet state of MA appearing in ∼100–200 ps, while MA-OCR interaction is evident from at least 5 ps (see Figure S7 in the Supporting Information). As such, we instead argue that the high concentrations of these formulations (i.e., the close proximity between MA and OCR molecules), as well as the complex environment of the formulation, could sufficiently increase the MA-OCR coupling to enable direct singlet-to-triplet energy transfer, from the S_1_ of MA to a triplet state in OCR, consequently resulting in the quenching of MA which we propose takes place within τ_3_ = 92 ± 0.7 ps.

We note here that this behavior could not plausibly be explained by the existence of ground state MA-OCR aggregates, since there is no evidence of these in the relevant UV/vis spectra (see Figure 1c). However, we cannot discard that the observed behavior would be due to a photoinduced aggregate, or ‘exciplex’ as we have previously described for MA and EHMC, even though we have no direct evidence of such species. In addition, the similarity between the ESA features at 350–450 nm in the TAS for both mixture formulation MAO and formulation O lends credibility to the energy transfer hypothesis. Regardless, all spectral features in the TAS for formulation MAO essentially disappear by 500 ps, apart from the small ESA at ∼375 nm which is assigned to MA photodynamics (see Section S2 of the Supporting Information for further discussion). This is indicative of a faster dissipation of energy in systems containing a mixture of MA and OCR, as opposed to systems where only MA is present.

In summary, while in formulations containing a mixture of MA and EHMC we find evidence of photochemistry with generation of an unidentified photoproduct (or exciplex), which is nonideal in a sunscreen formulation, in formulations containing MA mixed with OCR there is evidence of particularly efficient quenching of the excited states of MA, with no apparent generation of photoproducts resulting from the MA + OCR interaction. The main conclusions of our TEAS studies on thin solid film are summarized in Figure 4.

**Figure 4:**
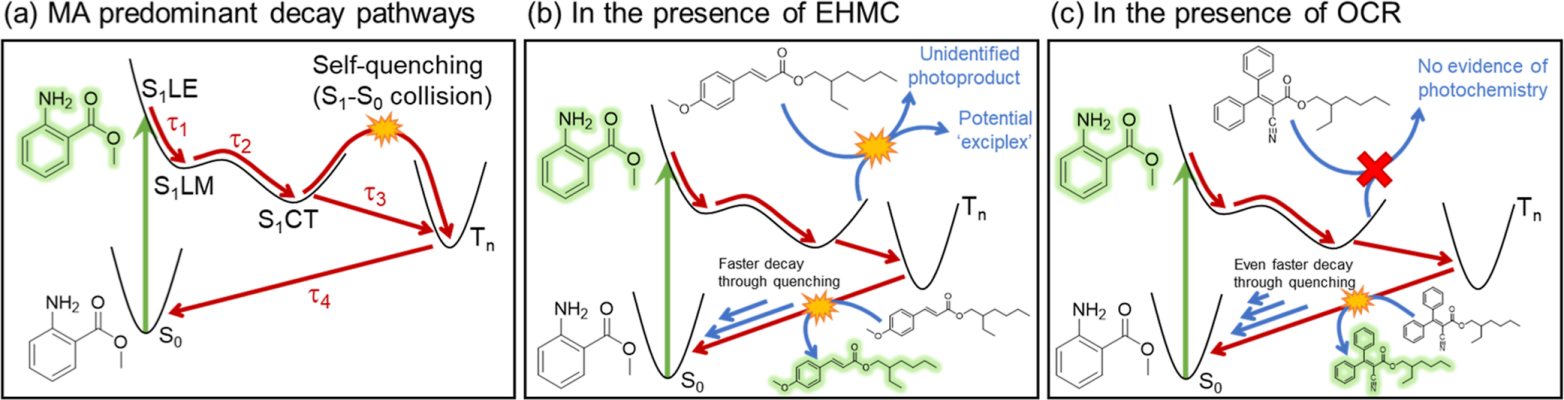
(a) Diagram depicting the proposed overall photodynamics for MA in the formulations under study, which is comparable to that observed in previous studies for dilute solutions (see main text for more details). (b,c) depict the main ways in which the MA photodynamics are affected by the presence of EHMC and OCR, respectively. We note here that, for the sake of simplicity, these diagrams suggest quenching by EHMC and OCR to take place via the triplet state of MA, while photochemistry with EHMC would take place from the excited singlet state of MA. However, this is not meant to discard the possibility that these processes would take place from other states; please see discussion in the main text for further details.

### Formulations on a Skin Mimic

Having made the step from solution to commercial sunscreen formulations, we then moved onward to study the same formulations applied to a skin mimic, to ascertain whether the skin surface had any discernible impact on the observed photodynamics. Briefly, we prepared the samples by applying each sunscreen formulation (MA_LC_, MA_HC_, MAE, and MAO) to a piece of substrate that mimics the thickness, viscoelasticity, chemical reactivity, and surface properties of human stratum corneum (VITRO–CORNEUM from IMS Inc.) and carried out TEAS measurements as before (see Methods).

Due to scattering from the skin mimic samples, we were unable to run full TEAS scans and instead acquired data only for a selected number of pump–probe time delays, as shown in Figure 5. Since no full scans were acquired, we were unable to extract meaningful time constants from these data sets; nevertheless, it is clear from the data presented in Figure 5 that the photodynamics observed on the skin mimic are essentially unaltered compared to those observed in formulations prepared onto CaF_2_ windows. We note, however, some minor differences that may indicate the influence of substrate on the observed photodynamics.

**Figure 5:**
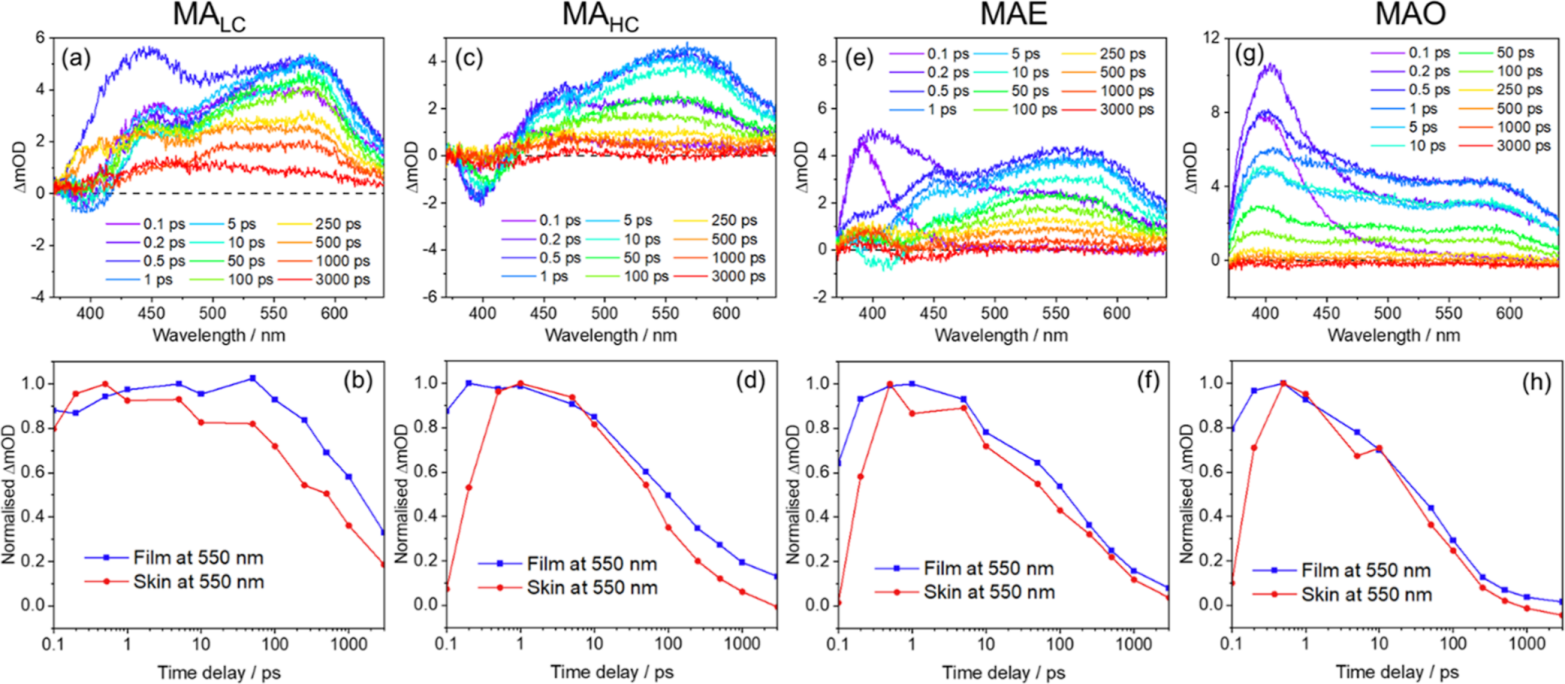
TEAS data obtained for each of the formulations under study applied to a skin mimic, namely (a) MA_LC_, (b) MA_HC_, (c) MAE, and (d) MAO, presented as transient absorption spectra at each pump–probe time delay (Δ*t*). Below are film vs skin comparisons between each trace at λ_pr_ = 550 nm for formulations (e) MA_LC_, (f) MA_HC_, (g) MAE, and (h) MAO. These comparisons are between the traces obtained for formulations prepared onto CaF_2_ windows and the equivalent ones for samples of formulations prepared onto pieces of skin mimic (see main text and Methods for further details). We note here that the ΔmOD signal for MA_HC_ is slightly lower than that of MA_LC_, which may seem contradictory; however, we note that our sample preparation methodology does not precisely control nor determine the exact amount of formulation applied to the skin at the irradiation spot, which voids direct comparison between ΔmOD values.

For formulations MA_LC_ and MA_HC_, the relatively weaker features at late-time Δ*t* indicate potentially faster decay of MA. The decay at λ_pr_ = 550 nm also seems to be slightly faster in skin (c.f. solid films on CaF_2_, see Figure 5b,d), but similar comparisons at other probe wavelengths, shown in Figure S8 in the Supporting Information, are much less obvious in this regard. While these data are not conclusive in determining faster decays for the formulations under study when applied to the skin mimic, it would be plausible to rationalize this hypothetical scenario considering the texture of the skin mimic, which creates ‘pockets’ in which formulation can gather, resulting in MA molecules being enclosed in a more restricted space, thus potentially promoting self-quenching (see earlier discussion) and facilitating faster relaxation of MA’s excited states.

In the case of the mixture formulations, MAE (Figure 5e,f) and MAO (Figure 5g,h), any differences are much less evident. In particular for MAO, the substrate has no apparent effect on the observed photodynamics. For formulation MAE, however, the weaker features of the long-time TAS (Figure 5e) might suggest a faster decay on skin, compared to solid films on CaF_2_. In previous discussion, we argued that the faster decay of MA in the presence of EHMC could be due to quenching or to the consumption of MA in its chemical reaction with EHMC. On skin, if the faster decay of spectral features attributable to MA were exclusively explained by consumption of MA in chemical reaction with EHMC, we might expect the photoproduct ESA (∼400 nm) to be relatively stronger than previously observed, which is not what we observe. We hypothesize that the surface of the skin mimic enhances quenching mechanisms (see earlier discussion), both between molecules of MA and between MA and EHMC, making them competitive toward MA + EHMC chemical reaction and ultimately resulting in faster energy dissipation from MA’s excited states, and less photoproduct being formed.

In summary, we have found that the photodynamics of the formulations under study are mostly unchanged when they are applied to the surface of a skin mimic substrate (c.f. when applied to a CaF_2_ window). Any minor changes that may be perceived from the data obtained for these samples points toward faster, more efficient energy dissipation following photoexcitation of these samples, potentially as a result of enhanced interactions facilitated by the confined spaces of the ‘pockets’ in the skin mimic’s surface texture, where formulation can gather once applied.

## Conclusion

This work has employed ultrafast laser spectroscopy techniques to study commercial sunscreen formulations and evaluate whether the observed photodynamics resemble those observed for dilute solutions of the individual UV filters. In general, we have found the photodynamics of isolated UV filters in formulation to be similar to those found in dilute solutions, but gathered evidence for significant photochemistry and quenching mechanisms not previously reported for mixtures of MA with either EHMC or OCR in formulation. Further, the photodynamical behavior observed for mixture formulations is clearly nonadditive, in the sense that it cannot be described by simply adding the behaviors of formulations containing only one of the UV filters. The appearance of these formulation-specific interactions is most likely due to the much higher concentrations of the UV filters present in these commercial formulations when compared to dilute solutions, as previously studied (i.e., ∼10^–3^ M). Importantly, throughout our work we find evidence of unidentified photoproducts which can be of concern for real-life application.

In addition, we collected photodynamical data for these formulations applied onto the surface of a skin mimic substrate. We have found the photodynamics of the formulations to be essentially unaltered when applied to the skin mimic.

To our knowledge, this is the first observation of energy transfer between UV filters in a commercial sunscreen formulation with femtosecond to picosecond resolution, as well as the first photodynamical study directly on sunscreen formulations applied onto a skin mimic. This level of insight into the photodynamic behavior of UV filters upon photoexcitation can inform the development of improved filters and formulations, thus facilitating a step-change in the sunscreen industry. In addition, this type of study is demonstrative in the relevance of ‘fundamental’ spectroscopy techniques and approaches to understanding real-life systems.

## Methods

No unexpected or unusually high safety hazards were encountered in any of the procedures described here. The formulas and preparation procedure for the sunscreen formulations studied in this work have been described in a separate publication,^14^ hence only a brief overview will be provided here (full formulas can be found in Section S1 of the Supporting Information). The sunscreen formulations prepared are oil-in-water emulsions, i.e. they consist of an oil phase dispersed in water, which acts as the continuous phase. The aqueous phase of these formulations was prepared by mixing a polymeric emulsifier (Pemulen EZ-4U, 0.15% (w/w)), a rheology modifier (Carbopol Ultrez 30, 0.2% (w/w)), and a humectant (Glucam E–20, 3% (w/w)) with water, under constant stirring with an overhead helix stirrer for ∼1 h at 700 rotations per minute (rpm). The oil phase was prepared separately by heating a mixture of an emulsifier (Glucamate SSE-20, 0.8% (w/w)) and UV filters to ∼50 °C until the mixture was clear. As it relates to UV filters, formulations MA_LC_ and MA_HC_ contained only MA at concentrations of 4% (w/w) and 19.5% (w/w), respectively. Formulations MAE and MAO contained mixtures of MA at 9.8% (w/w) with 9.7% (w/w) of either EHMC and OCR. The temperature of the aqueous phase was then raised to 50 °C before both mixtures were combined under constant stirring. The resulting mixture was homogenized using an Ultra-Turrax disperser (IKA-Werke GmbH & Co. KG, Staufen, Germany) rotating at 6000 rpm for 2 min, after which the mixture was allowed to cool to 30 °C under constant stirring. Finally, a preservative (Euxyl PE 9010) was added and the pH was adjusted to ∼6.5 with sodium hydroxide (NaOH, 18% w/w).

Thin solid films of each sunscreen formulation were prepared by applying approximately 0.01 g of each formulation onto a 1 inch diameter, 2 mm thick calcium fluoride (CaF_2_) window and pressing a second, 1 mm thick CaF_2_ window on top to spread the formulation between the two. The windows were then separated and allowed to dry, after which they were put back together and mounted on a Harrick’s cell. The two windows were once again pressed against each other to eliminate any air bubbles that might disrupt TEAS measurements. The quality of data was dependent on the quality of the films produced; to avoid photobleaching and to reduce noise in the data, the sample was translated 0.5 mm between each pump–probe time delay (Δ*t*, see below). Separately, samples of sunscreen formulations on a skin mimic were also prepared. In this case, a 1.5 × 1.5 cm sample of VITRO–CORNEUM (IMS Inc.) was rested on a 1 inch diameter, 2 mm thick calcium fluoride (CaF_2_) window, and 0.01 g of sunscreen was then applied to the skin mimic, using a gloved finger to spread the formulation on the skin mimic and to wipe away any excess cream. The sample was then allowed to dry and mounted on a Harrick’s cell prior to irradiation.

Transient electronic absorption spectroscopy (TEAS) measurements were carried out for the sunscreen formulations prepared as just described. A general overview of this experimental technique can be found in the review by Berera *et al.* ^27^ The TEAS setup used in these experiments has also been thoroughly described elsewhere;^28^ only the specific details pertaining to the reported studies will be provided here. The main laser system used in these experiments consists of a Mai Tai laser (Spectra-Physics) which seeds a commercial femtosecond (fs) Ti/sapphire regenerative amplifier (Spectra-Physics, Dual Ascend Pumped Spitfire Ace) which outputs 800 nm light with 12 W of peak power at 1 kHz repetition rate. A 2.5 W fraction of this output is seeded into an optical parametric amplifier (Topas-Prime with UV extension, Light Conversion) to generate the pump (λ_pu_) used in these experiments, centered at 335 nm, with a bandwidth of ∼500 cm^–1^ and a fluence of ∼3 mJ·cm^–2^ at the point of interaction with the sample. According to the previously reported absorption spectra of MA,^18^ EHMC,^16^ and OCR,^17^ as well as the UV/vis spectra presented in Figure 1 (see above), λ_pu_ = 335 nm is the peak absorbance of MA but will also significantly photoexcite EHMC and OCR. A separate 0.05 W fraction of the 800 nm fundamental beam was focused onto a vertically translated 2 mm thick CaF_2_ window to generate a broadband white light continuum (330–720 nm) that was used as the probe (λ_pr_) beam in these experiments. To avoid scattered pump light interfering with data collection, particularly for skin mimic samples, a wire grid polarizer was placed between the sample and the light detection apparatus (see below) to filter the pump light’s polarization, allowing transmission only of the white light (probe) polarization.

The TEAS data presented here is given in terms of changes in optical density (ΔOD), which are calculated from the measured probe intensities (as transmitted through the sample), collected using a fiber-coupled spectrometer (Avantes, AvaSpec-ULS1650F); ΔOD then corresponds to the difference in probe pulse intensities passing through sequentially excited and nonexcited sample, as prepared by a mechanical chopper in the pump beam path. Transient absorption spectra (TAS) were collected at several Δ*t* between λ_pu_ (photoexcitation) and λ_pr_ (interrogation), between −1 ps and 3 ns.

In order to extract dynamical information from the TEAS measurements, the data collected was globally fit using the Glotaran software package.^29^ The fitting algorithm used employs a sequential kinetic model (e.g., $A\overset{\tau_{1}}{\to }B\overset{\tau_{2}}{\to }C$ ) and convolves exponential decays with the Gaussian instrument response (IR, approximately ∼60 fs, see Section S3 of the Supporting Information, in particular Figure S4). In addition, the fitting algorithm also includes a third order polynomial to account for the fact that the TEAS data obtained in the present work is inherently chirped, i.e. Δ*t* = 0 is different for each probe wavelength (λ_pr_), due to group velocity dispersion artifacts.^30^ This fitting procedure allows the extraction of quantitative dynamical information by yielding time constants, τ, associated with each evolution associated difference spectra (EADS). The error associated with each extracted time constant, as presented in Table 1, is the error associated with the fit and should not be understood as an absolute measure of experimental error.

## Supplementary Material



## Notes

Section S1. Complete sample formulas; Section S2. TEAS for formulations containing only EHMC and OCR; Section S3. TEAS for reference formulations (no UV filters) and instrument response; Section S4. Transient absorption of MA’s triplet state in dilute solution; Section S5. Nonadditive behavior in mixture formulations; S6. Additional film vs skin comparisons (PDF)

## Abbreviations

CI: conical intersection
E: formulation containing only EHMC as the filter
EADS: evolution associated difference spectra
EHMC: ethylhexyl methoxycinnamate
ESA: excited state absorption
IC: internal conversion
ISC: intersystem crossing
MA: methyl anthranilate
MAE: formulation containing a mixture of MA and EHMC
MA_HC_: formulation with highest concentration of MA
MA_LC_: formulation with lowest concentration of MA
MAO: formulation containing a mixture of MA and OCR
O: formulation containing only OCR as the filter
OCR: octocrylene
SE: stimulated emission
SI: Supporting Information
TAS: transient absorption spectra
TEAS: transient electronic absorption spectroscopy
UV: ultraviolet
UV/vis: ultraviolet/visible
