# Supplementary material for: Ultrafast Spectroscopy of Commercial Sunscreen Formulations in Thin Films and on a Skin Mimic
Source: ACS Omega. 2026 Mar 11;11(11):17881–91. doi: 10.1021/acsomega.5c12445 (PMC13019394; doi:10.1021/acsomega.5c12445)
Supplement: Supplementary file 1 [file ao5c12445_si_001.pdf]

SUPPORTING INFORMATION

Ultrafast Spectroscopy of Commercial Sunscreen Formulations in Thin Films and on a Skin Mimic

Abigail L. Whittock,[1,†] Jack M. Woolley,[2,3,†] Juan Cébrian,[4] Vasilios G. Stavros,[5] and Natércia d. N. Rodrigues,[1,4,6,*]

[1] Department of Chemistry, University of Warwick, Gibbet Hill Road, Coventry, CV4 7AL, United Kingdom
[2] Department of Physics, University of Warwick, Gibbet Hill Road, Coventry, CV4 7AL, United Kingdom
[3] Warwick Centre for Ultrafast Spectroscopy, University of Warwick, Coventry, United Kingdom
[4] Lipotec SAU, The Lubrizol Corporation, Calle Isaac Peral, 17 Pol. Ind. Camí Ral, 08850 Barcelona, Spain
[5] School of Chemistry, University of Birmingham, Birmingham, B15 2TT, United Kingdom
[6] iBB-Institute for Bioengineering and Biosciences, Instituto Superior Técnico, Universidade de Lisboa, 1049-001 Lisboa, Portugal
[†] *These authors contributed equally to this work.*

KEYWORDS: photochemistry, photoprotection, spectroscopy, sunscreens, quenching

S1. Complete sample formulas

The sunscreen formulations under study in this work, which contain methyl anthranilate (MA), ethylhexyl methoxycinnamate (EHMC), and octocrylene (OCR) as ultraviolet (UV) filters, follow the same formulas and have been prepared in a similar fashion to that reported in a separate publication.[1] The formulas for MA_{LC} (sunscreen formulation containing the lowest concentration of MA), MA_{HC} (highest concentration of MA), MAE (MA+EHMC), and MAO (MA+OCR) are given in Table S1. Batches of 400 g of these formulas were prepared by first adding the aqueous phase ingredients to water one by one under constant stirring with an overhead helix stirrer. The resulting mixture was left to disperse for approximately 1 h under stirring at approximately 700 rpm. In a separate beaker, the ingredients of the oil phase were mixed with a magnetic stirrer bar and heated to approximately 50 °C until a clear mixture was achieved. The temperature of the aqueous phase was also raised to 50 °C, and the hot oil phase was then added to the aqueous phase under constant stirring. The resulting mixture was homogenized using an Ultra-Turrax disperser (IKA-Werke GmbH & Co. KG, Staufen, Germany) rotating at 6000 rpm for 2 min. The mixture was then transferred back to mechanical stirring and left to cool to approximately 30 °C, after which sodium hydroxide (NaOH, 18% w/w) was added until a pH ~6.5 was reached. The preservative (Euxyl PE 9010, Schülke and Mayr GmbH, Norderstedt, Germany) was added and, finally, the formulation was transferred to a glass container for storage before testing.

Table S1: Complete ingredient list and composition (formulas) of the formulations under study in the present work.

Phase	Raw Material	Supplier, City, Country	MA_{LC} *w/w %*	MA_{HC} *w/w %*	MAE *w/w %*	MAO *w/w %*
**Aqueous**	Deionized water	–	68.45	68.45	68.45	68.45
	Disodium EDTA	Ricardo Molina S.A.U., Baracelona, Spain	0.10	0.10	0.10	0.10
	PEMULEN* EZ-4U	Lubrizol Advanced Materials, Barcelona, Spain	0.15	0.15	0.15	0.15
	CARBOPOL* ULTREZ 30	Lubrizol Advanced Materials, Barcelona, Spain	0.20	0.20	0.20	0.20
	GLUCAM* E-20	Lubrizol Advanced Materials, Barcelona, Spain	3.00	3.00	3.00	3.00
**Oil**	GLUCAMATE* SSE-20	Lubrizol Advanced Materials, Barcelona, Spain	0.80	0.80	0.80	0.80
	SCHERCEMOL* LL	Lubrizol Advanced Materials, Barcelona, Spain	22.5	7.0	7.00	7.00
	Methyl Anthranilate (CAS 134-20-3)	Merck, Sigma-Aldrich, Madrid, Spain	4.00	19.50	9.80	9.80
	Ethylhexyl Methoxycinnamate (Neo Heliopan AV)	Symrise, Inc., Rennes, France	–	–	9.70	–
	Octocrylene (Neo Heliopan 303)	Symrise, Inc., Rennes, France	–	–	–	9.70
**Other**	Sodium Hydroxide (NaOH, 18%)	–	0.30	0.30	0.30	0.30
	Phenoxyethanol and ethylhexylglycerin (Euxyl PE 9010)	Schülke and Mayr GmbH, Norderstedt, Germany	0.50	0.50	0.50	0.50

Similarly, we prepared additional formulations containing only EHMC or OCR, to compare their photophysical behavior to that observed for these UV filters in neat solvent solutions (see section S2 below). In addition, a 'reference' formulation was prepared, containing no

UV filters, which was used to establish the instrument response of these experiments (see section S3 below). The formulas for these comparative/reference samples are given in Table S2.

Table S2: Complete ingredient list and composition (formulas) of the comparative/reference formulations under study in the present work.

Phase	Raw Material	Supplier, City, Country	E *w/w %*	O *w/w %*	Reference *w/w %*
**Aqueous**	Deionized water	–	68.45	68.45	68.45
	Disodium EDTA	Ricardo Molina S.A.U., Baracelona, Spain	0.10	0.10	0.10
	PEMULEN* EZ-4U	Lubrizol Advanced Materials, Barcelona, Spain	0.15	0.15	0.15
	CARBOPOL* ULTREZ 30	Lubrizol Advanced Materials, Barcelona, Spain	0.20	0.20	0.20
	GLUCAM* E-20	Lubrizol Advanced Materials, Barcelona, Spain	3.00	3.00	3.00
**Oil**	GLUCAMATE* SSE-20	Lubrizol Advanced Materials, Barcelona, Spain	0.80	0.80	0.80
	SCHERCEMOL* LL	Lubrizol Advanced Materials, Barcelona, Spain	16.8	16.8	26.5
	Methyl Anthranilate (CAS 134-20-3)	Merck, Sigma-Aldrich, Madrid, Spain	–	–	–
	Ethylhexyl Methoxycinnamate (Neo Heliopan AV)	Symrise, Inc., Rennes, France	9.70	–	–
	Octocrylene (Neo Heliopan 303)	Symrise, Inc., Rennes, France	–	9.70	–
**Other**	Sodium Hydroxide (NaOH, 18%)	–	0.30	0.30	0.30
	Phenoxyethanol and ethylhexylglycerin (Euxyl PE 9010)	Schülke and Mayr GmbH, Norderstedt, Germany	0.50	0.50	0.50

S2. TEAS for formulations containing only EHMC and OCR

To evaluate whether the "in-formulation" photodynamics of ethylhexyl methoxycinnamate (EHMC) and octocrylene (OCR) remain comparable to those observed in single-solvent conditions, we carried out transient electronic absorption spectroscopy (TEAS) experiments for formulations containing only EHMC or OCR as the UV filters (formulations E and O, respectively, see Table S2). The results of these TEAS experiments are presented in Figures S1 (MAE) and S2 (MAO). It is worth mentioning that the absorption spectra of these formulations (shown in the main manuscript, Figure 1) are in reasonable agreement with the absorption spectra reported for both EHMC and OCR in solution,[2,3] with only moderate broadening of the absorption features in both cases. Furthermore, the TEAS results for formulations E and O presented here were obtained for photoexcitation at λ_{pu} = 335 nm, for direct comparison with other experiments in this work (see main manuscript). These data were fit as described in the main manuscript (see Methods), using the Glotaran software package and employing a sequential kinetic model which convolves exponential decays with the Gaussian instrument response (see below). The results of this fitting are presented in Table S3.

These formulation results are in line with observations for both EHMC and OCR in solution. Peperstraete *et al.*[2] report on the photodynamics of dilute solutions (10^{-4} M) of EHMC dissolved in either cyclohexane or methanol. In their studies, the authors employed TEAS experiments and fitting methods in everything similar to the ones reported in this work. Peperstraete *et al.* assigned the shortest of the time constants extracted for each dataset (*i.e.* $\tau_1 < 1$ ps) to vibrational relaxation, followed by a $\tau_2 \sim 1.5$ ps time constant which the authors assigned to *E*/*Z* photoisomerization. In the formulation results we present here, we do not seem to capture the vibrational relaxation observable in solution, possibly due to the fact that we photoexcite EHMC at a lower energy (335 nm c.f. 290 or 308 nm in Peperstraete *et al.*'s work), making the vibrational relaxation less evident. In addition, the complexity of the sample's composition and/or its thickness or viscosity may also interfere with the vibrational relaxation of EHMC in these samples, precluding its observation in TEAS experiments. Nevertheless, we do extract a time constant of $\tau_1 \sim 1$ ps (see Table S3) which can plausibly be assigned to

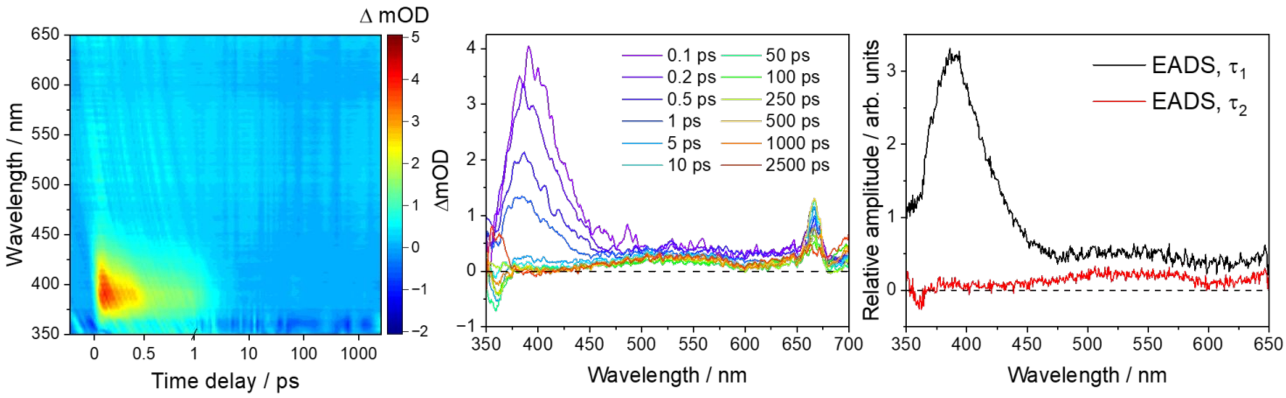

Figure S1: TEAS results for formulation E, i.e. sunscreen formulation containing only EHMC as the UV filter, photoexcited at λ_{pu} = 335 nm. On the left, TEAS data are presented as a false colour heatmap, with time being plotted linearly until 1 ps and on a logarithmic scale from then on. The same data are presented as transient absorption spectra at selected pump-probe time delays (Δt) in the middle graph. Finally, the graph on the right shows the EADS associated with each of the time constants extracted from globally fitting the TEAS data presented here (see main manuscript for further details).

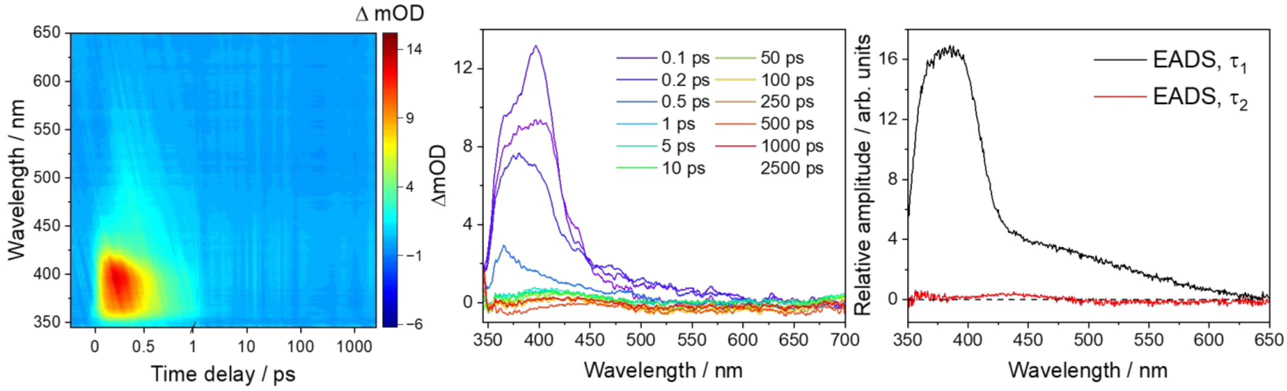

Figure S2: TEAS results for formulation O, i.e. sunscreen formulation containing only OCR as the UV filter, photoexcited at λ_{pu} = 335 nm. On the left, TEAS data are presented as a false color heatmap, with time being plotted linearly until 5 ps and on a logarithmic scale from then on. The same data are presented as transient absorption spectra at selected pump-probe time delays (Δt) in the middle graph. Finally, the graph on the right shows the EADS associated with each of the time constants extracted from globally fitting the TEAS data presented here (see main manuscript for further details).

E/Z photoisomerization of EHMC by comparison with the results and assignment by Peperstraete *et al.*[2] This observation confirms that E/Z photoisomerization takes place within the formulation environment as it does in solution.

Furthermore, Peperstraete *et al.* require a third time constant to appropriately fit their TEAS data for EHMC, which is longer than the temporal window of their experiments (*i.e.* $\tau_3 > 2$ ns). In our own TEAS data for EHMC in formulation, we also extract a long-lived time constant, $\tau_2 > 3$ ns (see Table S3). However, while the evolution associated decay spectra (EADS) associated with this long-lived time constant shows an excited state absorption (ESA) at ~300 nm for EHMC in solution,[2] in formulation the ESA feature is much broader and located at ~500-600 nm. In Peperstraete *et al.*'s work, the long-lived ESA is assigned to the Z-EHMC isomer, which is the photoproduct of the excitation of EHMC (naturally in its E-isomer). The stark spectral difference in the long-lived ESA observed in our TEAS results for EHMC in formulation suggest that, in addition to E/Z photoisomerization taking place, further reactions occur to generate a photoproduct other than the E-EHMC isomer, presumably due to the interaction of EHMC with other components of the formulation. Nevertheless, the overall photodynamics of EHMC in formulation are remarkably similar to those previously observed in solution. Finally, we note the positive feature at ~360 nm in the 2500 ps TAS (Figure S1, middle). We believe this feature is spurious and attribute this to instability in our white light generation at the shortest wavelength region. The peak visible at ~675 nm, on the other hand, corresponds to the second harmonic of the pump wavelength, which bleeds through from the TOPAS wavelength mixing process. This feature therefore has no physical meaning, in the sense that it does not reflect any photodynamical information.

The photodynamics of dilute solutions (10^{-2} M) of OCR dissolved in either cyclohexane or methanol were reported on by Baker *et al.*[3] In their work, the authors photoexcited OCR at λ_{pu} = 300 nm, which was proposed to access an ensemble of nearly isoenergetic excited states (S_n, with $n \geq 1$). The global fit of Baker *et al.*'s TEAS data for solutions of OCR yields four time constants, the first two of which are ultrafast (~80-130 fs) and are assigned by the authors[3] to internal conversion (IC) from higher lying excited electronic states down to less energetic ones. The authors then assign a time constant of $\tau_3 \sim 200$ fs to IC back to the ground electronic state (S_0) through a conical intersection (CI), with $\tau_4 \sim 0.8$-1.5 ps corresponding to vibrational relaxation on the S_0 state.[3]

In contrast, from our TEAS data for OCR in formulation we extract only two time constants (see Table S3). At first glance, this is in contrast with the work by Baker *et al.*;[3] however, it is entirely plausible that we are simply unable to capture as much vibrational detail from our TEAS experiments in formulation, compared to solution, for two main reasons. Firstly, our photoexcitation energies are relatively lower (300 nm in Baker *et al.*'s work *vs* 335 nm in the present study), likely accessing only the first electronic excited state (S_1) of OCR,[3] which explains the absence of the faster time constants assigned by Baker *et al.* to IC from higher lying electronic states. Secondly, it is plausible that the viscosity of the formulation environment (higher than that of solution) would hinder vibrational relaxation, making it less pronounced and therefore less likely to be captured in these experiments. The higher viscosity of the formulation environment is also likely to hinder the molecular motion associated with the CI through which IC to the ground state is suggested to take place,[3] effectively lengthening its associated time constant. Following this logic, we assign the first time constant ($\tau_1 = 420 \pm 60$ fs) extracted from our TEAS measurements for OCR in formulation to IC between the S_1 and S_0 states (c.f. ~200 fs in solution), with no further vibrational relaxation on the S_0 state being observable in our measurements.

We note here the appearance of a long-lived component ($\tau_2 > 3$ ns) in our TEAS measurements, which was not observed for OCR in solution. This long-lived component suggests the formation of a photoproduct upon irradiation of the formulation. Importantly, this long-lived component consists of a broad ESA centered approximately at 430 nm, which is clearly different from the long-lived ESA observed in formulations containing EHMC (centered at ~530 nm, see Figure S1). This difference suggests that, in each case, the photoproducts being formed are not the result of photoexciting the other (non-UV filter) formula ingredients, but rather are dependent on the interactions of the UV filter with the surrounding formulation environment. Despite this potential (and unidentified)

Table S3: Time constants extracted from the TEAS data for formulations E and O. The errors quoted are those associated with the fit, as produced by the fitting software package (reported to two standard deviations) or the instrument response, whichever is largest; more details in the Methods section of the main manuscript.

**Time constant**	Formulation E	Formulation O
**τ_1 / fs**	1005 ± 60	420 ± 60
**τ_2 / ns**	> 3	> 3

photoproduct, however, the photodynamics of OCR in formulation can be satisfactorily interpreted in light of previous findings for OCR in solution, suggesting no drastic alterations of OCR's photophysics in formulation vs solution, as was also concluded in the case of EHMC. These results support the present work's hypothesis that the photodynamics observed in solution remain relevant in complex commercial formulations containing UV-filters.

As a final remark on these measurements for EHMC and OCR in formulation, we note that all the studies presented and referred to, both here and in the main manuscript, are single-wavelength measurements which do not accurately represent the broad UV spectral range of real sunlight, a difference that can significantly impact the photodynamics of these UV filters. However, we have good reason to believe that, in these particular cases, the photodynamics will most likely be comparable under a broad UV spectral range. In the present work, we have photoexcited all samples with λ_{pu} = 335 nm and found that, in general, the photodynamics of the UV filters are comparable to those observed in other studies, for which the same UV filters were photoexcited at a range of other wavelengths. Specifically, in previous work by Rodrigues *et al.*,[4] MA was photoexcited at 300, 315, and 330 nm; in work by Peperstraete *et al.*,[2] EHMC was photoexcited at 290 and 308 nm; and, in work by Baker *et al.*,[3] OCR was photoexcited at 300 nm. Therefore, the combination of studies reported in the literature on the photodynamics of these UV filters covers the whole of the UVB region of the spectrum, as well as the highest energy portion of the UVA. Importantly, in all cases the photodynamics observed are comparable to those we report for photoexcitation of formulations containing these UV filters at 335 nm, as discussed above and in the main manuscript. As a result, we can conclude with some degree of confidence that the photodynamics taking place in formulations containing these UV filters, in isolation or in mixtures, upon photoexcitation with the broad UV spectral range of real sunlight would most likely be comparable to those reported in this work.

S3. TEAS for reference formulation (no UV filters) and instrument response

Transient electronic absorption spectroscopy (TEAS) was carried out for a reference formulation, i.e. one containing no UV filters (see Table S2), photoexcited at λ_{pu} = 335 nm. The resulting transient absorption spectra (TAS), shown in Figure S3, confirm that the reference formulation yields no observable photodynamics, apart from the expected time-zero artifacts which constitute the instrument response (see below). Hence, we conclude that all features observed in TEAS measurements of other formulations, as presented here and in the main manuscript, are due to the presence of UV filters (either due to their own photodynamics or to the interaction between them or with other components of the formulation).

The data presented in Figure S3 was also used to determine the instrument response of the TEAS experiments reported on in this work. Selected "slices" of these data sets at given probe wavelengths (namely, 350 nm, 400 nm, and 450 nm) were fit with a Gaussian function, as shown in Figure S4. The full width half maximum (FWHM) of the Gaussian fit is taken to be the instrument response; in this case, and

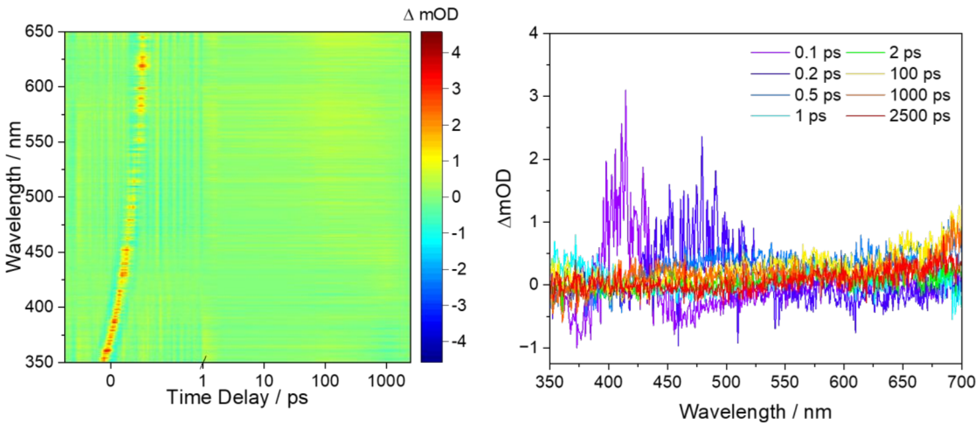

Figure S3: TEAS measurements obtained for a reference formulation, i.e. one containing no UV filters, according to the formula in Table S2, at λ_{pu} = 335 nm, shown as (left) a false colour heatmap, with time plotted linearly until 1ps and on a logarithmic scale from there on; (right) TAS traces at selected values of pump-probe time delays (Δt). These data confirm that the ingredients in the reference formulation have no observable photodynamics, and hence all features observed in TEAS measurements of other formulations are due to the presence of UV filters.

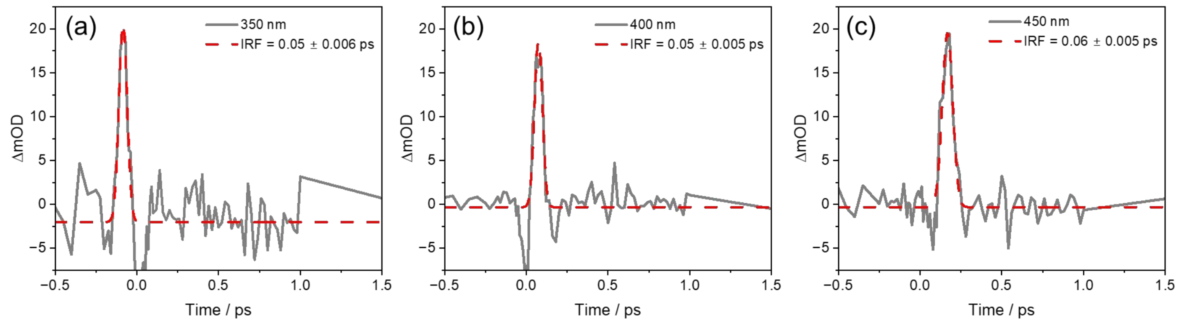

Figure S4: Instrument response measurements for the TEAS experiments performed in this work. The spectra presented correspond to "slices" of the TEAS data at selected probe wavelengths, namely (a) 350 nm, (b) 400 nm, and (c) 450 nm. Each spectrum (grey lines) was fit with a Gaussian (red dashed line), whose full width at half maximum (FWHM) is considered to be the instrument response.

following this approach, an instrument response of approximately 60 fs was determined. Analogous analyses have not been carried out for other probe wavelengths, particularly those further into the red edge of the probe spectrum, due to extremely low signal levels hindering meaningful analysis.

S4. Transient absorption of MA's triplet state in dilute solution

To complement the discussion in the main manuscript, we show here TEAS data for MA dissolved in cyclohexane (CHX) at a concentration of ~10^{-3} M, as previously published by Rodrigues *et al.*[4] We discuss in the main manuscript that the broad ESA centered at ~470 nm (spanning over ~400-500 nm), is likely due to the transient absorption of the triplet state of MA, since this spectral feature is very similar to that observed by Kikuchi *et al.*[5] for a close analogue of MA, Meradimate (or menthyl anthranilate). It is also based on this comparison with Kikuchi *et al.*'s work[5] that Rodrigues *et al.*[4] conclude that the final EADS extracted for MA in CHX (τ_3 > 2 ns, see green trace in Figure S5(b)) corresponds to the MA triplet state transient absorption. However, it is worth noting that while this EADS is required for appropriate fitting of the TEAS data for MA in CHX,[4] a corresponding ESA feature that could be attributed to an MA triplet state is not discernible in the TAS for MA in CHX (see Figure S5(a)). It can be concluded that, for MA in CHX, intersystem crossing (ISC) starts at the end of the temporal window of Rodrigues *et al.*'s experiments (Δt = 2 ns), so that while the MA triplet state transient absorption is not discernible in the TAS, the fitting procedure captures its contribution to the overall dynamics. This is in contrast with observations in formulation (both MA_{LC} and MA_{HC}, see Figure 2 in the main manuscript), where we clearly see TAS whose spectral features are attributable to MA's triplet state transient absorption well within the temporal window of these experiments. In fact, in formulation the broad ESA attributable to a triplet state absorption (~400-500 nm) is discernible from 250 ps onwards, approximately an order of magnitude earlier than in dilute solution.

One plausible explanation for this discrepancy is ISC in MA being more pronounced in formulation compared to dilute solution. This hypothesis aligns with our argument in the main manuscript that higher concentrations of MA facilitate self-quenching, explaining the faster decays in formulation vs solution, and in MA_{HC} vs MA_{LC} (see main manuscript). The comparison between MA in dilute solution vs formulation further suggests that MA self-quenching takes place via a mechanism whereby a photoexcited MA molecule (in the S_1 state) collides with a ground state MA molecule, transferring energy to the latter so that it accesses the S_1 state, from which ISC can then take place onto a triplet state. This mechanism explains both our observations: (i) that higher concentrations (i.e. more collisions) enhance self-quenching and therefore result in faster decay of MA's S_1 state, and (ii) that in formulation the MA triplet state transient absorption appears much earlier than in dilute solution, since the higher concentrations in formulation promote collisions between MA molecules,

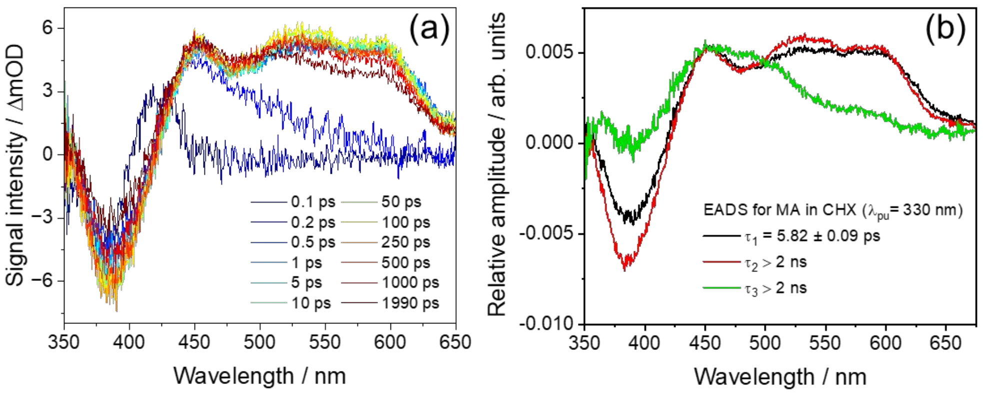

Figure S5: (a) TAS for MA in CHX at 10^{-3} M, photoexcited with λ_{pu} = 330 nm, at selected values of pump-probe time delay. (b) EADS extracted from fitting the TEAS data for MA as described in the main manuscript; also shown are the time-constants extracted from the same fitting. These data have been previously reported and discussed in reference 4.

which quench the S_1 state of MA to generate its triplet state (via ISC from the S_1 state). As such, our results not only show that the generation of the triplet state of MA is maintained in formulation (c.f. dilute solution), but also that self-quenching enhances triplet state generation.

S5. Non-additive TEAS behavior in mixture formulations

To further highlight the differences in photodynamics of mixture formulations – with MA being mixed with EHMC and OCR in formulations MAE and MAO, respectively – we compared the transient absorption spectra (TAS) for MAE and MAO with the sum of the corresponding TAS for formulations containing MA and EHMC or OCR separately. This comparison allows insight into whether the spectroscopic behavior of mixture formulations is merely additive (i.e. it is satisfactorily described as the sum of the TAS for MA and EHMC/OCR), or whether we see evidence of some interaction between these components in formulation. To clarify, the 'added spectra' shown in Figures S6 and S7 correspond to the sum of the TAS obtained at corresponding pump-probe time delays (Δt) for formulations MA_{LC} and E or O (containing EHMC or OCR, respectively, as the only UV filters), with no other data processing apart from the sum having been carried out. In this sum, we opted for using the TAS for MA_{LC} (rather than MA_{HC}) because the concentration of MA in MA_{LC} is closer to the concentration of MA in the mixture formulations: 4% (w/w) vs 9.8% (w/w), respectively (c.f. 19.5% (w/w) in MA_{HC}). However, we did confirm that repeating this exercise with the TAS for MA_{HC} yields comparable results.

As evident in Figure S6, at Δt = 10 ps the TAS for formulation MAE is mostly comparable to that of the added spectra i.e., the sum of the TAS at Δt = 10 ps for formulations MA_{LC} and E. This is in line with the conclusion discussed in the main manuscript that, immediately following photoexcitation of formulation MAE, the photodynamics are dominated by EHMC's behavior. However, the stimulated emission (SE) feature at 400 nm, attributable to MA (see main manuscript), is more prominent in the added spectra than in the equivalent TAS for formulation MAE. This might be indicative of faster decay of MA's excited state in the presence of EHMC (evidenced by the faster disappearance of the SE feature), as we have argued in the main manuscript.

For larger values of Δt, the effect of the presence of EHMC in the mixture is much more apparent. At Δt = 50 ps, the added TAS retains the strong SE feature at 400 nm, as well as the broad ESA feature at ~450-650 nm, while in the TAS for the mixture formulation MAE the SE feature is no longer visible, and the broad ESA feature is significantly weaker. Furthermore, while the added spectra is virtually unchanged at Δt = 500 ps, the TAS for MAE already shows a strong new ESA at ~375 nm, which we argue in the main manuscript is the result of photochemistry between MA and EHMC, since we do not observe an equivalent feature in any of the TAS for formulations containing only MA or EHMC separately. Finally, at Δt = 2500 ps, the SE feature has almost disappeared from the added spectra, and the

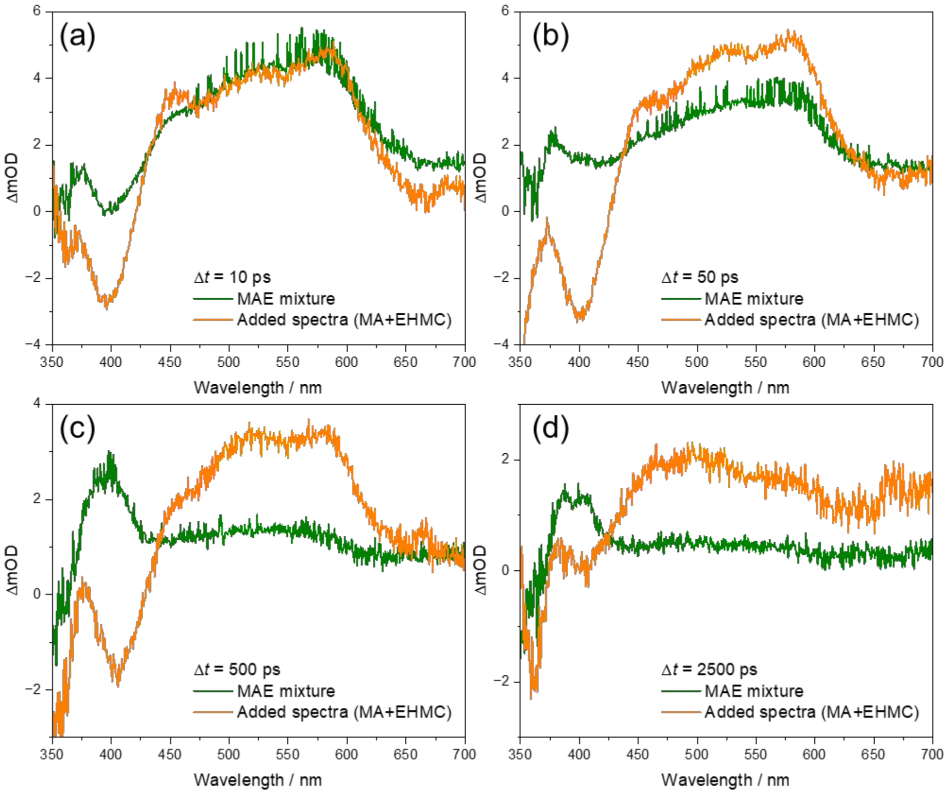

Figure S6: TAS for MAE mixture formulation (green lines) at Δt = (a) 10 ps, (b) 50 ps, (c) 500 ps, and (d) 2500 ps. Also shown for comparison are 'added spectra' i.e., spectra corresponding to the sum of the TAS for formulations MA_{LC} and E at each given Δt. These comparison spectra clearly show that the photodynamics of the mixture formulation MAE are not suitably described by an additive effect of MA and EHMC's photodynamics but are instead the result of an interaction between these UV filters.

ESA feature is greatly decayed; in the TAS for MAE, the broad ESA feature between ~450-650 nm is completely decayed, and the photoproduct ESA at 375 nm remains. From this comparison, we can conclude that the photodynamics of the mixture formulation, MAE, cannot be fully described by assuming an additive effect of MA and EHMC's photodynamics, but instead is best explained by considering the interaction – energy transfer leading to photochemistry – between MA and EHMC. As further discussed in the main manuscript, we conclude that the faster decay of spectral features attributable to MA in the presence of EHMC (i.e. in the TAS for formulation MAE) is likely due to both quenching and photochemical reaction between MA by EHMC, as further discussed in the main manuscript.

A similar approach was employed for formulation MAO, which contains a mixture of MA and OCR; the TAS at selected Δt were compared to corresponding spectra produced by adding the TAS of formulations MA_{LC} and O (containing only MA and OCR, respectively) at the same values of Δt. The added MA+OCR data is only significantly different from the behavior observed in MA-only formulations at very early times (e.g. Δt = 0.5 ps in Figure S7), since the photodynamics of OCR are extremely fast and complete by 5-10 ps (see Section S2 above and reference 3); as such, the added spectra mostly resemble MA-only formulations from as early as 5 ps and for the remainder of the temporal window of these experiments. This is in stark contrast with observations for formulation MAO, whose TAS consist of a single, broad ESA feature which could initially (Δt = 0.5 ps) be described as a combination of ESA features from MA (~450-650 nm) and OCR (~350-450 nm). However, a broad ESA persists until at least 50 ps, when the decay of similar features in OCR-only samples is complete by approximately 10 ps. Alongside this persistence, a decay of the broad ESA attributable to MA (~450-650 nm) is evident in the TAS for formulation MAO between 5-50 ps. From the next evaluated TAS (Δt = 500 ps) all spectral features have completely decayed, except for a minor ESA at ~375 nm which is also present in the added spectra and is accounted for by MA photodynamics.

The behavior just described suggests a strong interaction between MA and OCR, evident immediately after photoexcitation. We rule out the possibility of this interaction being due to a ground-state aggregate, since the UV/Vis absorption spectra for these formulations (presented in Figure 1 of the main manuscript) do not reveal an additional absorption band for formulation MAO that would indicate the presence of species other than MA and OCR separately. As such, two potential explanations for the observed behavior remain: (i) the generation of a photoinduced aggregate, or (ii) energy transfer between MA and OCR leading to quenching of photoexcited MA. While we see no direct evidence of a photoinduced aggregate, we cannot discard that the spectral features present in the TAS for formulation MAO might be due to absorption from such a species. However, we argue that the observed behavior is most likely due to energy transfer mechanisms between the two UV filters, since energy transfer between these two molecules is reported in the literature.[6] Matsumoto *et al.*[6] point out that singlet-to-singlet energy transfer between these molecules, leaving two potential mechanisms for this energy transfer, as discussed in the main manuscript: (i) triplet-to-triplet energy transfer, which is unlikely since the evidence of MA-OCR interaction is present at much earlier times than the ESA corresponding to the triplet state of MA; (ii) direct

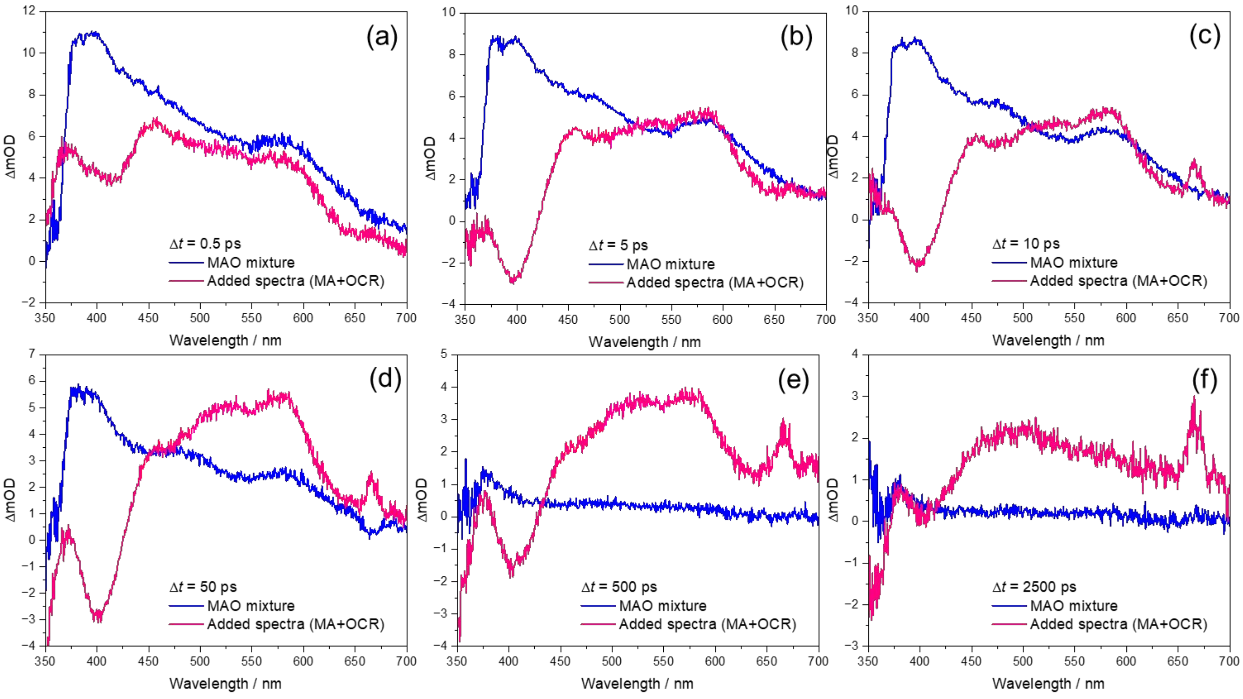

Figure S7: TAS for formulation MAO (blue lines) at Δt = (a) 10 ps, (b) 50 ps, (c) 500 ps, and (d) 2500 ps. Also shown for comparison are 'added spectra' *i.e.*, spectra corresponding to the sum of the TAS for formulations MA_{LC} and O at each given Δt. These comparison spectra clearly show that the photodynamics of the mixture formulation MAO are not suitably described by an additive effect of MA and OCR's photodynamics, but are instead the result of an interaction between these UV filters. The peak visible at ~675 nm for the MA+OCR added spectra, which comes from the MA datasets, corresponds to the second harmonic of the pump wavelength, which bleeds through from the TOPAS wavelength mixing process. This feature therefore has no physical meaning, in the sense that it does not reflect any photodynamical information.

singlet-to-triplet energy transfer which, albeit a forbidden transition, we argue becomes possible to the high concentration and complex environment of the formulations under study. Notably, the ESA corresponding to the triplet state of MA has been found to persist beyond the temporal windows of these experiments (Δt > 3 ns) in formulation and in solution (see main manuscript and reference 4; respectively), while it completely disappears by 500 ps in the presence of OCR. Unlike the case previously discussed, for formulation MAE, no ESA feature that could be attributed to a photoproduct is visible, hence the possibility of significant photochemistry taking place between MA and OCR is discarded in this case. As previously discussed, a long-lived ESA at ~375 nm is present in both the added spectra and the TAS for MAO, indicating some photochemistry due to MA's isolated behavior. We note here that global analysis of the data for formulation MAO yields a lifetime of τ_4 = 2 ± 0.03 ns for the EADS where this ~375 nm is present (see Table 1 in the main manuscript), which compares to the > 3 ns lifetime for the final EADS for both formulations MA_{LC} and MA_{HC} (see main manuscript for further details). However, since the EADS associated with τ_4 > 3 ns for formulations MA_{LC} and MA_{HC} include both ESA features (at ~375 nm and the one attributable to MA's triplet state), the difference in lifetimes might be explained by the convolution of these features in the data for formulations MA_{LC} and MA_{HC}.

S6. Additional film vs skin comparisons

As stated in the main manuscript, due to significant scattering we were not able to collect full TEAS datasets for skin mimic samples. Consequently, we were also not able to fit the data and extract time constants to compare with those extracted for thin solid films (on CaF_2 windows). Instead, for skin mimic samples we collected data at only selected pump-probe delays, and compare these traces with equivalent ones for the thin solid film data, as shown in Figure S8. In the main manuscript, we make this comparison for traces at a probe

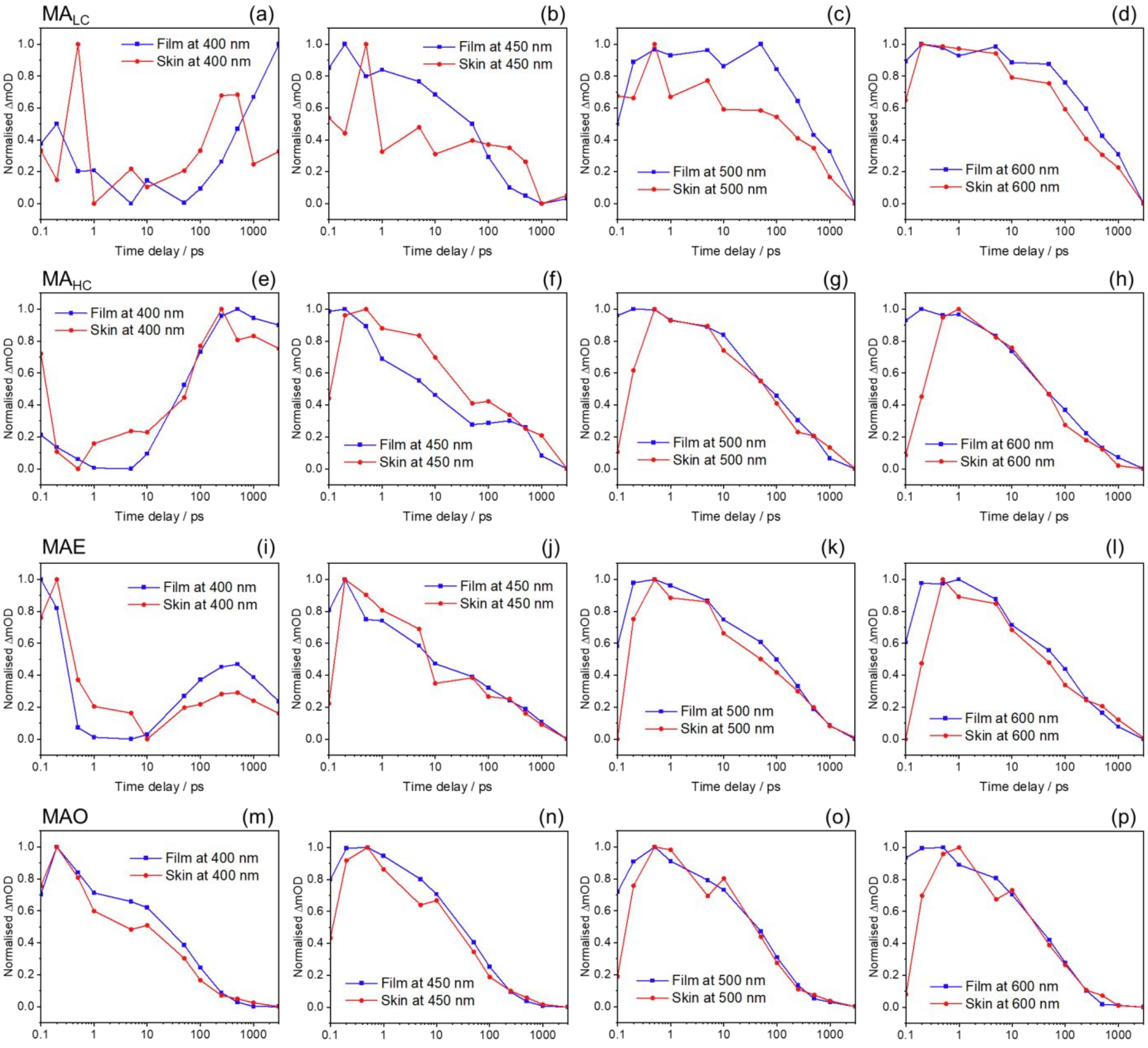

Figure S8: Additional film vs skin comparisons between each trace at several probe wavelengths (400nm, 450 nm, 500 nm, and 600 nm, from left to right) for formulations (a-d) MA_{LC}, (e-h) MA_{HC}, (i-l) MAE, and (m-p) MAO. These comparisons are between the traces obtained for formulations prepared onto CaF_2 windows and the equivalent ones for samples of formulations prepared onto pieces of skin mimic (see main manuscript for further details).

wavelength of 550 nm (see Figure 5), and argue that these comparisons hint at potentially faster decays in the skin mimic, when compared to thin solid films, particular for MA-only formulations (not so much for mixture formulations MAE or MAO). The comparisons in Figure S8 seem to loosely support this conclusion, even though the differences between the two datasets are almost imperceptible. As discussed in the main manuscript, these comparisons offer no conclusive evidence that the decays are accelerated (or significantly altered, in any way) when the formulations under study are applied to a skin mimic; however, if that were to be the case, it could plausibly be rationalized in light of the skin mimic surface texture (see main manuscript for further discussion).

References

(1) Rodrigues, N. d. N.; Cebrián, J.; Montané, A.; Mendez, S. Intermolecular Interactions and In Vitro Performance of Methyl Anthranilate in Commercial Sunscreen Formulations. *AppliedChem* **2021**, *1* (1), 50–61. https://doi.org/10.3390/appliedchem1010005.

(2) Peperstraete, Y.; Staniforth, M.; Baker, L. A.; Rodrigues, N. D. N.; Cole-Filipiak, N. C.; Quan, W.-D.; Stavros, V. G. Bottom-up Excited State Dynamics of Two Cinnamate-Based Sunscreen Filter Molecules. *Physical Chemistry Chemical Physics* **2016**, *18* (40), 28140–28149. https://doi.org/10.1039/C6CP05205C.

(3) Baker, L. A.; Horbury, M. D.; Stavros, V. G. Ultrafast Photoprotective Properties of the Sunscreening Agent Octocrylene. *Opt. Express* **2016**, *24* (10), 10700. https://doi.org/10.1364/OE.24.010700.

(4) Rodrigues, N. D. N.; Cole-Filipiak, N. C.; Horbury, M. D.; Staniforth, M.; Karsili, T. N. V.; Peperstraete, Y.; Stavros, V. G. Photophysics of the Sunscreen Ingredient Menthyl Anthranilate and Its Precursor Methyl Anthranilate: A Bottom-up Approach to Photoprotection. *J. Photochem. Photobiol. A Chem.* **2018**, *353*, 376–384. https://doi.org/10.1016/j.jphotochem.2017.11.042.

(5) Kikuchi, A.; Shibata, K.; Kumasaka, R.; Yagi, M. Excited States of Menthyl Anthranilate: A UV-A Absorber. *Photochemical & Photobiological Sciences* **2013**, *12* (2), 246–253. https://doi.org/10.1039/c2pp25190f.

(6) Matsumoto, S.; Kumasaka, R.; Yagi, M.; Kikuchi, A. Triplet–Triplet Energy Transfer between UV Absorbers in Solutions at Room Temperature. *J. Photochem. Photobiol. A Chem.* **2017**, *346*, 396–400. https://doi.org/10.1016/j.jphotochem.2017.06.020.
